# Sanhuang Fukang oil alleviates X-ray-induced skin injury by reducing inflammation and apoptosis: an *in vivo* study

**DOI:** 10.3389/fphar.2025.1684426

**Published:** 2026-01-06

**Authors:** Ruochen Zhu, Mandi Lin, Wei Deng, Xintong Jian, Chiwei Chen, Xinlei Shi, Milin Lai, Mei Huang

**Affiliations:** 1 The First Affiliated Hospital of Guangzhou University of Chinese Medicine, Guangzhou, Guangdong, China; 2 The Laboratory of Orthopaedics and Traumatology of Lingnan Medical Research Center, Guangdong Clinical Research Academy of Chinese Medicine, Guangzhou University of Chinese Medicine, Guangzhou, China; 3 The Second Affiliated Hospital of Guangzhou University of Traditional Chinese Medicine, Guangzhou, Guangdong, China

**Keywords:** radiation dermatitis, ROS, MAPK, PI3K-AKT, traditional Chinese medicine, apoptosis

## Abstract

**Background:**

Radiation dermatitis is a significant dose-limiting toxicity of radiotherapy that compromises treatment efficacy and patient quality of life. Although topical anti-inflammatory agents and emollients provide symptomatic relief, they fail to address the underlying pathophysiological mechanisms. SHFKO, a botanical extract with documented antioxidant and anti-inflammatory properties, has been used in traditional medicine but its molecular mechanisms of action remain poorly characterized. This study systematically investigated the therapeutic potential of SHFKO in mitigating radiation-induced skin injury through integrated chemical, molecular, and histopathological analyses.

**Methods:**

Ultra-performance liquid chromatography (UPLC) was used to characterize the bioactive components of SHFKO. A mouse model of radiodermatitis was monitored for cutaneous manifestations and healing. Expression levels of inflammatory mediators (MMP13, IL-6, IL-1β), antioxidant enzymes (catalase, HO-1, SOD-1, Nrf2), and apoptotic regulators (cleaved-caspase 3, Bcl-2) were measured using RT-PCR and Western blotting to assess radiation-induced oxidative stress and apoptosis. The effects of SHFKO on MAPK/NF-κB/PI3K-AKT signaling pathways were evaluated by Western blotting and immunohistochemistry.

**Results:**

UPLC analysis confirmed a diverse bioactive profile of SHFKO. *In vivo* experiments demonstrated significant attenuation of cutaneous inflammation and enhanced tissue regeneration. RT-PCR and Western blot analyses revealed dose-dependent anti-inflammatory effects of SHFKO, with the high-dose treatment achieving effects comparable to the reference drug, MFC (p > 0.05). Mechanistic studies revealed pathway-specific modulation, with anti-radiodermatitis effects primarily mediated by inhibition of MAPK/PI3K-AKT phosphorylation rather than the NF-κB pathway. Immunohistochemical staining confirmed that SHFKO normalized the radiation-induced upregulation of MMP13, IL-6, IL-1β, integrin β1, and CXCL9. Additionally, SHFKO exhibited anti-apoptotic activity, which accelerated cutaneous repair.

**Conclusion:**

This study demonstrated significant therapeutic effects of SHFKO in treating radiodermatitis, characterized by accelerated healing of acute radiation-induced cutaneous injuries. The multi-targeted mechanisms involved apoptosis inhibition, reduction of pro-inflammatory mediators, MAPK/PI3K-AKT pathway inhibition, and restoration of ROS homeostasis. These findings suggest SHFKO as a promising candidate for clinical development in radiation dermatitis treatment, offering a multi-target therapeutic approach distinct from conventional approaches.

## 1 Introduction

Radiation therapy is a cornerstone treatment in modern oncology. Using high-energy ionizing radiation to induce DNA damage in cancer cells, it serves as a cost-effective approach for malignant neoplasm treatment (Bertholet et al., 2021). During radiotherapy, both tumor cells and bystander cells undergo radiation-induced damage to DNA and cellular components. The resulting genetic alterations produce biological effects that are dependent on factors such as cumulative dose and fractionation schedules (Rosenthal et al., 2019). Radiotherapy elicits inflammatory responses of varying intensity, manifesting as edema and capillary leakage. Prolonged radiotherapy produces fibrosis and vascular endothelial injury, leading to end-organ dysfunction (Liu and Wang, 2021). These pathological changes, including radiation dermatitis, oral mucositis, and radiation pneumonitis, are clinically recognized as distinct adverse effects (Liu and Wang, 2021).

Radiation dermatitis represents the most prevalent complication of radiotherapy, affecting up to 95% of patients undergoing radiation treatment (López et al., 2002). Of these patients, 85% develop moderate to severe skin damage, particularly those with cervical neoplasms and breast cancer (Liu and Wang, 2021; López et al., 2002; Chin et al., 2017; Tao et al., 2017). These adverse cutaneous reactions predominantly manifest in the thoracic and head-neck regions, typically emerging 2–3 weeks post-treatment initiation, peaking at the conclusion of therapy, and gradually subsiding within 2–3 weeks after treatment completion (De Ruysscher et al., 2019). Based on onset timing, radiation dermatitis is classified into acute and chronic manifestations (De Ruysscher et al., 2019). Acute radiation-induced dermatitis (ARD), characterized by an early onset and pronounced symptomatology, has garnered significant attention in recent years. This condition typically develops within 90 days of treatment initiation, with severity correlating directly with the cumulative radiation exposure. Higher radiation doses are associated with more severe tissue damage (Ryan, 2012). Persistent acute radiation dermatitis may progress to chronic radiation dermatitis, which is characterized by hyperpigmentation, epidermal thinning, dermal atrophy, and telangiectasia (Wilson et al., 2022). These cutaneous alterations are typically irreversible, and the associated pain, recurrent wound formation, and restricted mobility can significantly compromise the quality of life of patients (Wilson et al., 2022; Rossi et al., 2014). The severity of cutaneous damage increases patient morbidity, with reports indicating that severe radiation dermatitis requires treatment interruption in approximately 58.1% of cases, thus potentially compromising therapeutic efficacy (Spałek, 2016). In advanced stages, radiation-induced mutagenesis may lead to secondary cutaneous malignancies (Ramseier et al., 2020). Consequently, the prevention and early intervention of radiation dermatitis are important considerations in clinical radiotherapy practice.

Currently, there is no standardized protocol for the prevention and treatment of acute radiation dermatitis. While corticosteroid ointments, such as mometasone furoate cream (MFC), have received guideline endorsement for their prophylactic and therapeutic efficacy, their clinical use remains limited due to associated adverse effects (Behroozian et al., 2023). Alternative therapeutic agents generally lack robust empirical support, and contemporary medical interventions are often prohibitively expensive for widespread implementation. In traditional Chinese medicine (TCM), radiation dermatitis is categorized within the paradigm of burns and ulcerations, and is attributed to the superficial invasion of pathogenic factors such as heat toxins. The TCM therapeutic approach predominantly uses topical preparations that cool blood, detoxify, clear heat, dry dampness, and promote tissue regeneration. The topical medicinal oil San Huang Fu Kang oil (SHFKO) is an in-house preparation developed at the First Affiliated Hospital of Guangzhou University of Chinese Medicine. This preparation is based on *Coptis chinensis* Franch. [*Ranunculaceae; Coptidis Rhizoma*], Rheum officinale Baill. [*Polygonaceae; Radix et Rhizoma Rhei*], Phellodendron amurense Rupr. [Rutaceae; Cortex Phellodendri], Potash Alum [Alumen] and Synthetic Borneol [*Borneleum syntheticum*] in equal proportions (1:1:1:1:1), macerated in olive oil. Preliminary clinical trials of breast cancer patients have demonstrated the ability of SHFKO to ameliorate symptoms associated with acute radiation dermatitis, including pain, erythema, and pruritus, while promoting lesion resolution (refer to Supplementary Material S1). The overall response rate reached 91.7%, and showed non-inferiority with a medically prescribed radioprotective agent, a topical superoxide dismutase. However, the molecular mechanisms underlying the therapeutic effects of SHFKO in acute radiation-induced skin injury remain to be elucidated.

This investigation aimed to evaluate the effects of SHFKO on mouse models and cellular models of ARD, and further explore its potential reparative mechanisms.

## 2 Methods and results

### 2.1 Reagents and materials

Primary antibodies targeting ERK (cat: #BF8004), phosphorylated ERK (cat: #AF1015), MEK (cat: #AF6385), phosphorylated MEK (cat: #AF8035), JNK (cat: #AF6318), phosphorylated JNK (cat: #AF3318), P38 (cat: #BF8015), phosphorylated P38 (cat: #AF4001), IκB-α (cat: #AF5002), SOD-1 (cat: #AF5144), HO-1 (cat: #AF5393), catalase (cat: #DF7545), Nrf2 (cat: #AF0639), MMP13 (cat: #AF5355), IL-6 (cat: #DF6087), cleaved-caspase 3 (cat: #AF7022), cleaved-caspase 9 (cat: #AF5240), Bcl-2 (cat: #AF6139), and IL-1β (cat: #AF5103) were procured from Jiangsu Affinity Biosciences Co., Ltd. (Jiangsu, China). Molecular biology reagents, including an RT-qPCR kit (cat: AG11708) and Evo M-MLV reverse transcription kit (cat: AG11705), were acquired from Accurate Biology. A Bicinchoninic Acid (BCA) protein assay kit (BL521A) was obtained from Biosharp Life Sciences (Anhui, China). RIPA lysis buffer (P0013B) and a phosphatase and protease inhibitor cocktail (P1045) were purchased from Beyotime Biotechnology (Shanghai, China). Chemical standards for rhein (lot no. 10701), chrysophanol (lot no. 5118), emodin (lot no. 13675), aloe emodin (lot no. 14346), physcion (lot no. 14291), berberine hydrochloride (lot no. 9434), palmatine (lot no. 9491) epiberberine (lot no. 14315), coptisine (lot no. 12516), and phellodendrine (lot no. 13904) were purchased from Shanghai Standard Technology Co., Ltd. (Shanghai, China). HPLC grade acetonitrile was purchased from Merck (Darmstadt, Germany). Water was prepared using a water purification system (RODI-200B1, RSJ Scientific Instruments Co., Ltd., Xiamen, China).

Female C57BL/6J mice (approximately 8 weeks of age) were sourced from the Laboratory Animal Center of Guangzhou University of Chinese Medicine (ethics committee approval number: 20241227010). Using a randomized allocation protocol, the animals were divided into sham, model, and experimental groups, with the latter further stratified into an MFC group and two SHFKO groups (administered at low and high concentrations). Following a 7-day acclimatization period under standardized laboratory conditions, the animals underwent radiation-induced dermatitis modeling.

SHFKO was prepared as follows: *Coptis chinensis* (25 g), *Rheum officinale* (25 g), *Phellodendron amurense* (25 g), potash alum (25 g), and borneol (25 g) were ground into a fine powder. The powdered botanical drug mixture was then added to 250 mL of olive oil preheated to 70 °C–80 °C and thoroughly mixed using mechanical stirring. The mixture was then sealed in an airtight container and allowed to macerate for 1 week at room temperature. The preparation was filtered through sterile gauze to remove particulate residue, yielding the final medicated oil product. For experimental applications, the concentrated oil was serially diluted with olive oil to achieve the desired concentrations. This standardized protocol ensured consistent extraction of bioactive compounds while maintaining the physicochemical stability of the formulation.

### 2.2 UHPLC/MS-MS analysis of SanHuang FuKang oil

For analysis, 5 mL of SHFKO was added to ethanol (25 mL) and sonicated (250 W, 40 kHz) for 30 min. The supernatant was collected and filtered through a 0.22-µm filter. The reference standards were dissolved in methanol to produce 1 mg/mL solutions. The mixed standards were prepared by mixing 100 µL of each standard and diluting to 2 mL with methanol.

An ACQUITY UPLC HSS T3 column (100 mm × 2.1 mm, 1.8 µm) was used to separate the sample at a temperature of 35 °C. Mobile phase A was H2O with 0.1% formic acid and mobile phase B was acetonitrile. The flow rate was 0.2 mL/min. The gradient was as follows: 0–4 min, 30%–35% B; 4–6 min, 35%–75% B; 6–10 min, 75%–95% B; and 10–14 min, 95% B. The injection volume was 5 μL.

Full MS and data-dependent tandem mass spectrometry 2 (ddMS2) in both positive and negative ion mode were used to acquire full scan MS and MS2 data of the samples. A scan mass range of 100–1,200 Da was selected. High resolution full-scan MS and MS2 data were collected at a resolving power of 70,000 and 17,500, respectively. HCD collision energy was 30%, 40%, and 50%. The temperature of the capillary was 350 °C, and the spray voltage was 3 kV in negative ion mode and 3.5 kV in positive ion mode. The flow rate of the sheath gas (N2) and auxiliary gas (N2) were 50 and 10 arb, respectively. The data obtained were analyzed with Thermo Xcalibur 4.0.27 Qual Browser (Thermo Fisher Scientific).

After collecting MS data of the test solution, structural characterization and confirmation of metabolites were performed using Xcalibur software (version 4.0, Thermo Fisher scientific Inc.).

### 2.3 Establishment of the animal model

In accordance with previously established protocols, animals were administered 1% pentobarbital sodium for anesthesia. Hair removal from the dorsal skin was performed using an electric clipper to minimize mechanical trauma. Following depilation, each mouse was carefully inspected to confirm the absence of micro-cuts, abrasions, or visible skin irritation. Only mice with intact skin barriers, as determined by visual examination, were advanced to the irradiation procedure. This quality control step ensured that baseline skin integrity was uniform across all experimental groups. The subjects were properly immobilized, and appropriate shielding was applied to protect all areas outside the designated treatment zone. The animals were then positioned to ensure precise exposure of the depilated area to the radiation field (Sheng et al., 2019; Korpela et al., 2014). With the exception of the control cohort, radiation-induced dermatitis was established in all experimental animals using a medical linear accelerator (Elekta, Beijing, China). A single-fraction of X-ray irradiation was delivered under standardized parameters (total dose, 45 Gy; beam energy, 6 MeV; dose rate, 600 cGy/min; source-to-skin distance, 100 cm). Following a 7-day acclimatization period with *ad libitum* access to food and water, animals were randomly allocated into a control group, a model group, and experimental groups, which comprised the MFC group and SHFKO groups (low-dose and high-dose SHFKO groups). All groups except the model group received daily topical administration for 4 consecutive weeks and animal welfare was monitored daily for body weight, general condition, and activity level. No mortality or systemic toxicity occurred during the study. Upon completion of the treatment period, animals were placed in a euthanasia chamber and exposed to carbon dioxide at a fill rate of 10%–30% of the chamber volume per minute. After complete loss of consciousness and cessation of respiration, CO_2_ exposure was maintained for an additional 1–2 min to confirm death, followed by tissue collection and subsequent analyses. This experimental protocol was approved by the Institutional Animal Care and Use Committee, and all procedures were conducted in accordance with the principles of laboratory animal welfare and ethics.

### 2.4 Evaluation and quantification of cutaneous lesions

Sequential photographic documentation of the affected cutaneous regions was obtained at 48-h intervals using standardized digital imaging (Nikon camera system). Lesions were assessed in accordance with previously validated scoring criteria to confirm that the model was successfully established (Korpela et al., 2014). The severity of cutaneous manifestations was evaluated based on multiple parameters, including the presence and extent of erythema, desquamation, ulceration, and crust formation. The lesion area and healing kinetics were quantified weekly using ImageJ software to objectively measure the affected surface area and calculate wound healing rates.

### 2.5 ELISA

Mice were deeply anesthetized using CO_2_ and blood was collected. The blood samples were centrifuged at 2,000 rpm and 4 °C for 20 min to isolate the supernatant. Standard wells, sample wells, and blank control wells were prepared. Standard wells and sample wells were loaded with 50 μL of serially diluted standards and test samples, respectively, while blank control wells were loaded with 50 μL of the sample dilution buffer. Subsequently, 100 μL of horseradish peroxidase-conjugated detection antibody was added to each well. The plate was sealed and incubated at 37 °C for 45 min. After aspirating the liquid, each well was washed five times with 350 μL of wash buffer (20-s incubation per wash). Next, 50 μL of substrate A and B were added to each well, followed by a 15-min incubation at 37 °C in the dark. Reactions were terminated by adding 50 μL of stop solution, and optical density values were measured at 450 nm.

### 2.6 Histological analysis of cutaneous tissue

Excised cutaneous specimens were fixed in a 4% paraformaldehyde solution and processed using a standardized histological protocol. The tissue was processed using an increasing ethanol gradient and paraffin infiltration, and then was embedded. Tissue sections were prepared using a microtome, followed by deparaffinization and rehydration using a decreasing ethanol gradient. Specimens were subjected to hematoxylin and eosin staining according to standard protocols. The stained sections were mounted with a neutral mounting medium and subjected to microscopic examination and analysis using bright-field microscopy.

### 2.7 Immunohistochemical staining

Skin tissue specimens were sectioned to a thickness of 4 μm and subjected to a sequential protocol comprising xylene-based deparaffinization, antigen retrieval, and successive incubation with primary and corresponding secondary antibodies. The specimens were then counterstained with DAPI. Stained sections were visualized, and photomicrographs were obtained using optical microscopy.

### 2.8 Western blot

Total protein was extracted from skin tissue specimens using RIPA lysis buffer, and protein concentrations were quantified using a bicinchoninic acid (BCA) assay. Target proteins were separated through sodium dodecyl sulfate-polyacrylamide gel electrophoresis and subsequently transferred onto polyvinylidene fluoride membranes. After blocking with bovine serum albumin, the membranes were washed three times using tris-buffered saline with Tween 20 and incubated overnight with specific primary antibodies, followed by incubation with the corresponding secondary antibodies. Target proteins were ultimately visualized using enhanced chemiluminescence detection reagents.

### 2.9 Quantitative real-time PCR (RT-PCR) analysis

Total RNA was isolated from skin tissue specimens using an RNA extraction kit according to the manufacturer’s instructions, followed by reverse transcription to complementary DNA (cDNA) using a cDNA synthesis kit. The relative expression levels of target genes were assessed using SYBR Green-based quantitative PCR performed on a Bio-Rad CFX96 real-time PCR detection system. *β-Actin* served as the internal reference gene, and the relative gene expression was quantified using the 2^−ΔΔCT^ method. The primer sequences for the analyzed genes are presented in Table 1 (Luo et al., 2023; Zhuang et al., 2023).

**TABLE 1:** Primer sequences.

Genes	Forward primer (5′–3′)	Reverse primer (5′–3′)
*β-actin*	TGC​TAT​GTT​GCC​CTA​GAC​TTC​G	GTT​GGC​ATA​GAG​GTC​TTT​ACG​G
*MMP13*	GCC​ACC​TTC​TTC​TTG​TTG​AGT​TG	GAC​TTC​TTC​AGG​ATT​CCC​GCA
*IL6*	ACT​CAC​CTC​TTC​AGA​ACG​AAT​TG	CCA​TCT​TTG​GAA​GGT​TCA​GGT​TG
*IL1B*	TTC​GAC​ACA​TGG​GAT​AAC​GAG​G	TTT​TTG​CTG​TGA​GTC​CCG​GAG

### 2.10 Statistical analysis

All statistical analyses were performed using GraphPad software, and the results are expressed as mean ± standard deviation (SD). One-way analysis of variance (ANOVA) was used for intergroup comparisons, while multiple group comparisons were conducted using non-parametric tests. Statistical significance was defined as p ≤ 0.05.

## 3 Results

### 3.1 UPLC–HRMS analysis of chemical metabolites of San Huang Fu Kang oil

**FIGURE 1:**
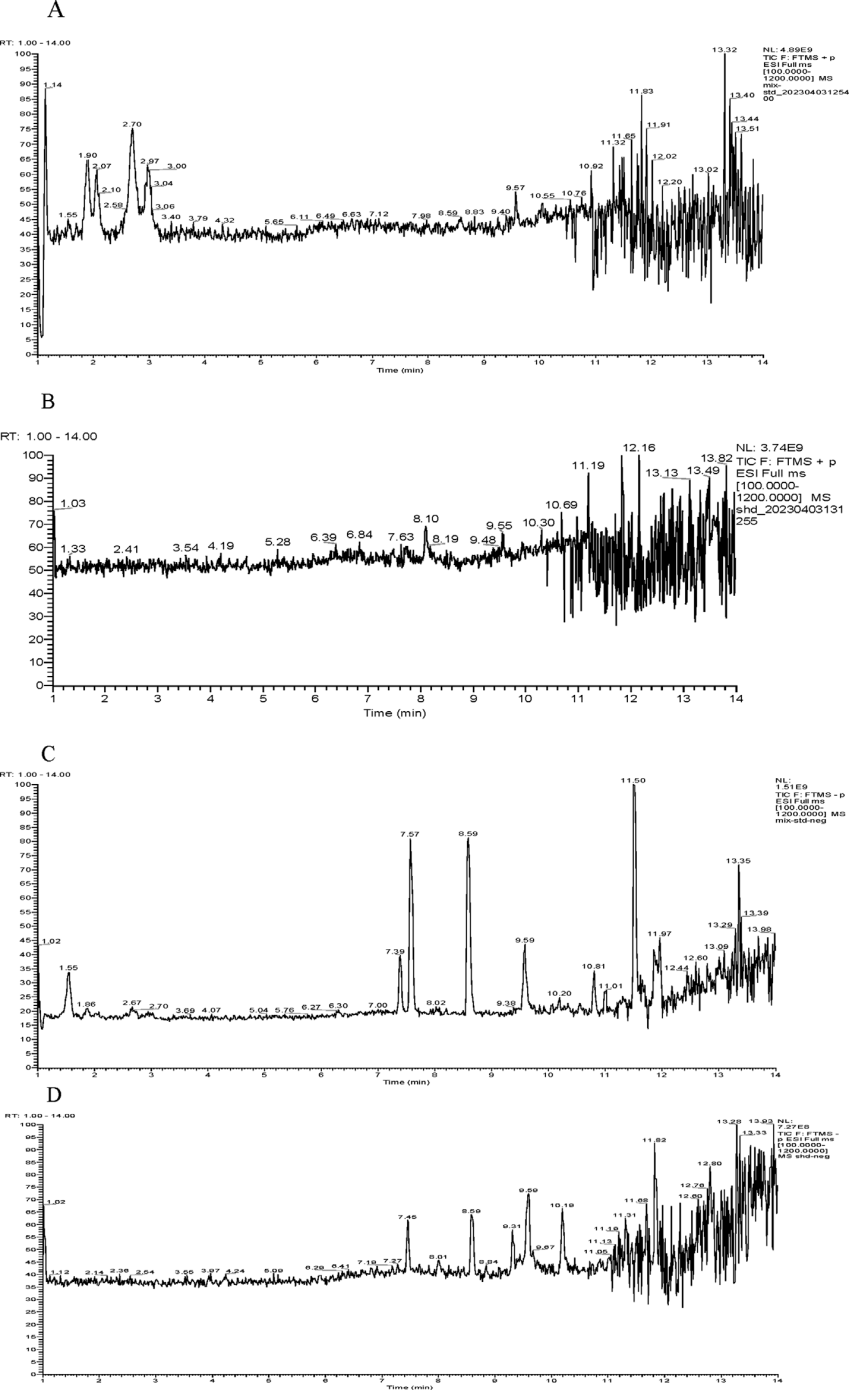
Total ion chromatography of SanHuang FuKang oil. **(A)** Positive mode of the reference standards, **(B)** positive mode of the sample solution, **(C)** negative mode of the reference standards, and **(D)** negative mode of the sample solution.

To identify the metabolites of SHFKO, a sample solution was analyzed by UHPLC–MS/MS. The total ion chromatograms are shown in Figure 1. After processing with Xcalibur software and comparing with reference standards and a databank, as shown in Table 2, the significant metabolites were identified in the test solution.

**TABLE 2:** Tentatively identified compounds in SanHuang FuKang oil.

No.	Name	Formula	Ion mode	Theoretical value	Retention time	Measured value	Error	Fragmentation peak
1	Tetrahydroprotoberberine alkaloid	C20H24NO4 ^+^	M ^+^	342.169	1.03	342.1682	−5.274	-
2	Lotusine	C19H24NO3^+^	M^+^	314.1751	1.09	314.1734	−5.316	-
3	Catechin	C15H14O6	M-H^−^	289.0718	1.12	289.07098	−0.7611	203.06964, 123.04332, 109.02783
4	Epicatechin	C15H14O6	M-H^−^	289.0718	1.12	289.07098	−0.8129	203.06964, 123.04332, 109.02783
5	Phellodendrine^a^	C20H24NO4	M^+^	342.1705	1.14	342.16843	−4.544	192.1011, 177.0778
6	3-O-Feruloylquinic acid/5-O-feruloylquinic acid	C17H20O9	M-H^−^	367.1044	1.2	367.10312	−0.914	191.0547, 173.0441, 155.0334, 134.0357
7	Menisperine	C21H26NO4	M^+^	356.1862	1.2	356.1842	−4.028	-
8	(+) N-Methylcorydine	C21H25NO4	M+H^+^	356.1851	1.2	356.18367	−8.596	
9	Feruloylquinic acid isomer	C17H20O9	M-H^−^	367.1033	1.21	367.10353	0.203	193.0478, 173.0448, 134.03550
10	Rhein 8-glucoside	C21H18O11	M-H^−^	445.0776	1.24	445.07855	3.28033	
11	Protocatechualdehyde	C7H5O3	M-H^−^	137.0245	1.35	137.02301	−1.902	108.0199, 81.0329
12	N-Methyltetrahydrocolumbamine	C21H26NO4	M^+^	356.1862	1.44	356.1841	−4.365	-
13	Tetrahydropalmatine	C21H25NO4	M+H^+^	356.1849	1.44	356.18364	−8.68	
14	Epicatechin gallate	C22H18O10	M-H^−^	441.0827	1.46	441.08182	−0.8048	
15	2-O-Cinnamoyl-beta-D-glucose/1-O-cinnamoyl-beta-D-glucose	C15H17O7	M-H^−^	309.0980	1.59	309.09743	1.426	
16	Jatrorrhizine	C20H20NO4	M^+^	338.1387	1.78	338.1372	−4.361	323.1134, 308.0884, 294.1111, 280.0930
17	Amurenlaetone A	C17H19O9	M-H^−^	367.1052	1.82	367.103	−1.747	191.0547, 134.0357, 117.0333
18	2-(2′-Hydroxypropyl)-5-methyl-7-hydroxychromone-	C13H14O4	M-H^−^	233.0819	1.83	233.08122	−0.7079	
19	Ferulic acid	C10H9O4	M-H^−^	193.0506	1.86	193.04945	−0.131	178.0261, 149.0593, 134.0361
20	4,5-Dihydro-9,10- dimethoxybenzo[g]-1,3- benzodioxolo[5,4- a]quinolizinium	C20H18NO4	M^+^	336.1220	1.87	336.12146	−4.684	320.0904, 292.0958
21	Columbamine	C20H20NO4	M+H^+^	338.138	1.88	338.1372	−4.361	
22	Epiberberine^a^	C20H18NO4	M^+^	336.1223	1.88	336.12152	−4.506	320.0904, 292.0958
23	6-Hydroxyrumicin-8-O-glucoside	C19H21O9	M-H^−^	393.1186	1.91	393.11835	0.868	
24	(6aS)-1,2,10,11-Tetramethoxy-6,6- dimethyl-5,6,6a, 7-tetrahydro-4H-dibenzo [de, g] quinolinium	C22H28NO4	M^+^	370.2018	1.91	370.20028	−2.714	
25	N-Methyltetrahydropalmatine	C22H28NO4 +	M^+^	370.2009	1.91	370.19971	−4.254	
26	Coptisine^a^	C19H13NO4	M+H^+^	320.0913	2.05	320.09074	−6.533	
27	1-O-Galloyl-2-O-cinnamoyl-glucose/1-O-galloyl-6-O-cinnamoyl-glucose	C22H21O11	M-H^−^	461.1089	2.09	461.10834	1.089	
28	6-Dehydroxylaccaicacid D-glucoside	C22H20O11	M-H^−^	459.0927	2.23	459.09213	−0.126	
29	Palmatine	C21H22NO4	M^+^	352.1549	2.71	352.1528	−4.443	336.1211, 322.1054, 308.1266, 294.1104
30	Berberine^a^	C20H18NO4	M^+^	336.123	3.01	336.1218	−3.67	321.0975, 320.0904, 306.0749, 292.0957
31	Torachrysone-8-O-β-D-glucopyranoside	C20H24O9	M-H^−^	407.1348	3.43	407.134	−0.5158	245.08144, 230.05742, 215.03336
32	Chrysophanol-8-O-β-D-glucoside	C21H20O9	M-H^−^	415.1035	3.46	415.10309	1.762	253.05003,225.05363
33	Chrysophanol-1-O-β-D-glucoside	C21H19O9	M-H^−^	415.1035	3.8	415.10291	1.328	253.05003, 225.05363
34	γ-Hydroxybutenolide	C19H34O15	M-H^−^	501.1814	4.23	501.17692	−10.528	457.1857, 413.1925, 395.1894, 371.1869
35	Emodin 8-O-β-D-(6′-O-malonylglucoside)	C24H21O13	M-H^−^	517.0977	4.54	517.09802	0.683	
36	Torachrysone 8-O-acetylglucoside	C22H26O10	M-H^−^	449.1452	4.79	449.14468	−0.2115	245.08134, 230.05740
37	Acetyl-emodin-O-glucoside	C23H22O11	M-H^−^	473.1084	4.92	473.10892	2.287	269.0453, 225.0537
38	Physcion 8-β-D-glucoside	C22H22O10	M-H^−^	445.1140	5.41	445.11313	−0.7751	283.06055
39	Acetyl-chrysophanol-O-glucoside	C23H20O10	M-H^−^	457.1135	5.55	457.11377	0.124	
40	Luteolin	C15H10O6	M-H^−^	285.0382	6.04	285.03989	−2.004	257.0460, 211.0397
41	Citreorosein	C15H10O6	M-H^−^	285.0412	6.05	285.03983	−0.298	267.0659, 239.0706
42	γ-Fagarine	C13H12NO3	M+H^+^	230.0803	6.18	230.0804	−3.563	215.0574, 200.0340
43	13-Methoxyljatrorrhizine	C21H22NO5	M^+^	368.1492	6.24	368.1476	−4.371	321.1206, 277.0949, 251.0796, 207.0536
44	Rutaevin	C26H30O9	M-H^−^	485.1812	6.32	485.18079	−1.887	441.1904, 397.2003
45	Physcion-8-O-beta-D-(6′-O-acetyl)glucopyranoside	C24H24O11	M-H^−^	487.1237	6.65	487.12384	−0.4106	
46	Rutaevin isomer	C26H31O9	M+H^+^	487.1959	6.84	487.1946	−3.323	
47	6-Dehydroxylaccaicacid D	C16H10O6	M-H^−^	297.0399	6.9	297.04004	0.42082	253.05009, 225.05450
48	13-Hydroxypalmatine	C21H22NO5 +	M^+^	368.1485	7.34	368.14746	−4.86	321.1203, 277.0933
49	Aloeemodin^a^	C15H10O5	M-H^−^	269.0455	7.36	269.04523	0.8548	240.04202, 211.03871, 183.04364
50	Limonin	C26H31O8	M+H^+^	471.2011	7.47	471.19953	−3.405	425.1939, 407.1831, 161.0591
51	Rhein^a^	C15H8O6	M-H^−^	283.0248	7.58	283.02405	−0.7597	
52	8-Oxyberberine/berberastine	C20H18NO5	M+H^+^	352.1179	7.86	352.1163	−4.769	337.0926, 322.0695, 308.0901, 294.0746
53	Kaempferol-3-O-rutinoside	C27H29O15	M-H^−^	593.1516	8.01	593.1572	11.925	121.0278
54	Obacunone	C26H30O7	M+H^+^	455.2060	8.19	455.2048	−3.58	209.1641, 114.0917
55	N-Methylflindersine	C15H16NO2	M+H^+^	242.1169	8.21	242.1167	−3.73	200.0688, 188.0699
56	Obaculactone derivative	C26H28O8	M-H^−^	467.1706	8.34	467.17047	−1.436	409.1349
57	3-O-Feruloylquinic acid glucoside	C23H29O14	M-H^−^	529.1557	8.43	529.1497	−10.322	
58	Emodin^a^	C15H9O5	M-H^−^	269.0455	8.54	269.0453	3.346	241.04974, 225.05438, 197.05946
59	N-Methylhigena-7-O-glucopymnoside	C23H28NO8	M-H^−^	446.1809	8.83	446.17777	−9.43	
60	3-Carboxy-4-hydroxyphenoxy glucoside	C13H15O9	M-H^−^	315.0711	8.85	315.07483	8.68	
61	Procyanidin B1	C30H25O12	M-H^−^	577.1352	9.07	577.13452	−0.1473	
62	2-Methyl-5-carboxymethyl-7-hydroxychromone	C12H10O5	M-H^−^	233.0455	9.61	233.04529	1.24439	
63	Chrysophanol^a^	C15H9O4	M-H^−^	253.0506	9.61	253.05043	3.536	225.0544
64	Magnoflorin	C20H24NO4	M+H^+^	342.1705	10.01	342.1684	−4.631	
65	Physcion^a^	C16H11O5	M-H^−^	283.0612	10.07	283.06036	0.919	
66	8-Methyltetrahydropalmatine	C22H28NO4 +	M^+^	370.2005	10.22	370.19998	−3.525	
67	Linoleic acid	C18H32O2	M-H^−^	279.2333	11.84	279.23328	1.169	193.0484, 175.0923
68	Hexadecanoic acid	C16H31O2	M^−^	255.2333	12.61	255.23201	−3.697	237.2222, 162.1711
69	Gallic acid	C7H6O5	M-H^−^	169.0142	12.77	169.01376	0.355	125.02269, 107.04867, 79.95592

^a^ Identified with reference compounds.

### 3.2 Effects of SHFKO and MFC on histopathological changes during lesion repair

A histopathological analysis revealed varying degrees of inflammatory improvement across all study groups during the lesion repair process. At week 2, the model group exhibited hyperkeratosis, epidermal hypertrophy, and enhanced inflammatory cell infiltration. The MFC group demonstrated a therapeutic response with ameliorated dermatitis symptoms, while the SHFKO group showed alleviated hyperkeratosis, reduced epidermal hypertrophy, and attenuated inflammatory cell infiltration.

**FIGURE 2:**
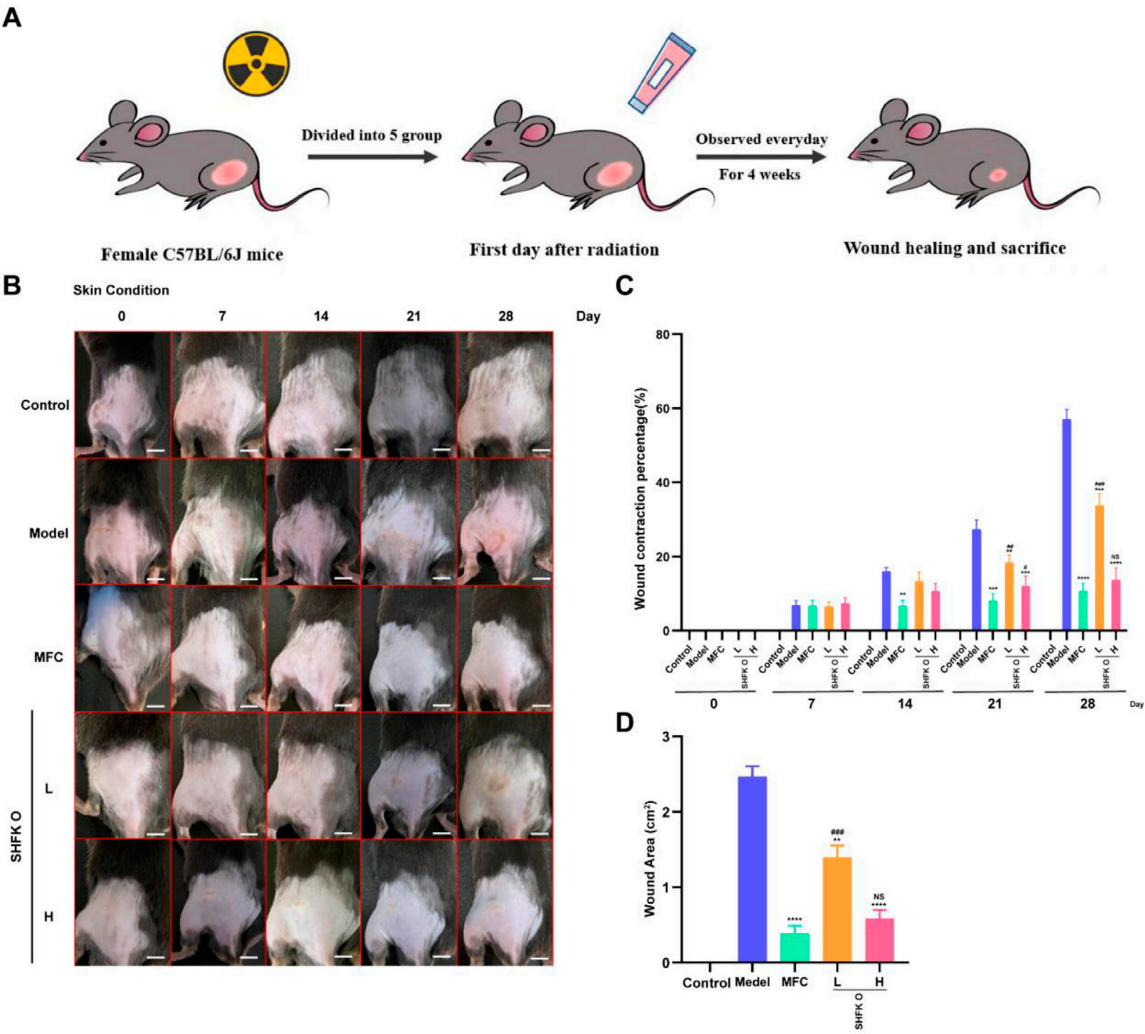
Assessment of the cutaneous lesions and the repair process. **(A)** Representative images depicting the progression of skin lesions in each experimental group at weekly observation intervals. **(B)** Dermatitis severity scores across treatment groups. The MFC group exhibited significant therapeutic cutaneous repair effects from week 2 onwards. Notably, from week 3, the SHFKO group demonstrated comparable effects to those of the MFC group in terms of enhanced wound healing and attenuated inflammatory responses. **(C,D)** Comparative analysis of skin lesion development and repair status across all experimental groups. MFC vs model: *p < 0.05, **p < 0.01, ***p < 0.001; SHFKO vs model: #*p* < 0.05, ##*p* < 0.01, ###*p* < 0.001; NS: not significant (n=8). Scale bars: 500 μm.

By week 4, while the model group continued to display severe pathological changes, the MFC group achieved an optimal therapeutic response with lesion repair. Both the low- and high-concentration SHFKO groups demonstrated comparable therapeutic outcomes as the MFC group, characterized by normal keratinization, appropriate epidermal stratification, and minimal inflammatory cell infiltration (Figures 2A,B). Furthermore, the results shown in Figures 2C,D indicate that the MFC and SHFKO groups exerted their therapeutic effects by ameliorating inflammatory cell infiltration while maintaining normal cutaneous characteristics. Notably, in week four, the SHFKO groups, and particularly the high concentration group, exhibited therapeutic effects comparable to those of the MFC group.

### 3.3 SHFKO promotes lesion repair and alleviates the inflammatory response by suppressing inflammatory mediators and regulating ROS levels

**FIGURE 3:**
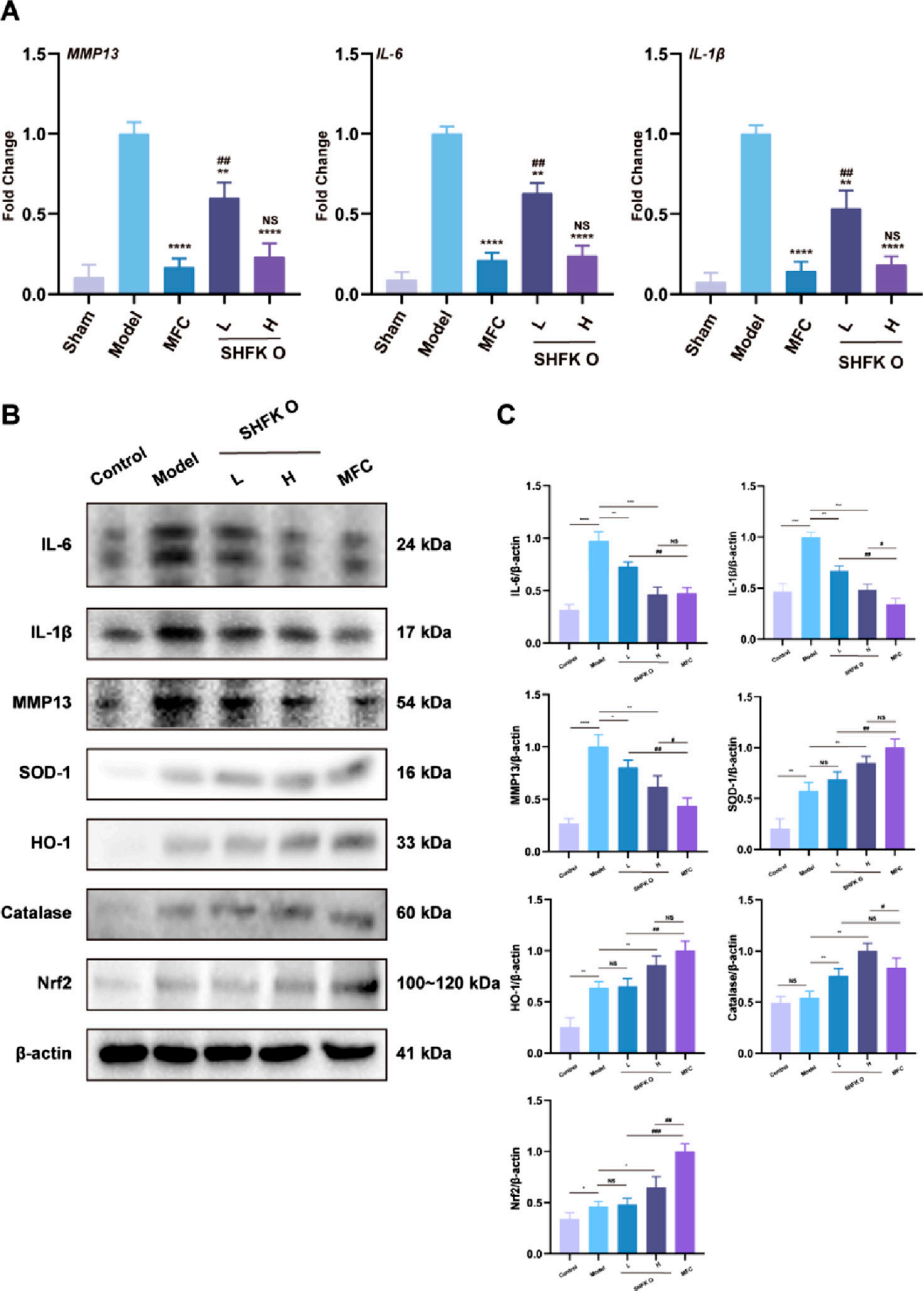
SHFKO modulates inflammatory mediators and ROS-related protein expression. **(A)** RT-PCR analysis demonstrated the regulatory effects of SHFKO on the inflammatory mediators *MMP13*, *IL-6*, and *IL-1β*. **(B,C)** Western blot analysis revealed the dual functionality of SHFKO in suppressing inflammatory mediator expression while simultaneously modulating the levels of antioxidant proteins (SOD-1, HO-1, catalase, Nrf2). MFC vs. model and SHFKO vs. model: *p < 0.05, **p < 0.01, ***p < 0.001; SHFKO vs. MFC: #*p* < 0.05, ##*p* < 0.01, ###*p* < 0.001; NS, not significant (n = 8).

We investigated the modulatory effects of SHFKO on the relative expression of inflammation-associated genes (*MMP13*, *IL-6*, *IL-1β*) and ROS-related proteins (SOD-1, HO-1, catalase, Nrf2) through RT-PCR and Western blot analyses, respectively. As shown in Figure 3A, X-ray radiation exposure increased inflammatory marker expression in the model group. However, intervention with either MFC or SHFKO significantly downregulated *MMP13*, *IL-6*, and *IL-1β* gene expression. Notably, the high-concentration SHFKO group demonstrated comparable anti-inflammatory effects as the MFC group (Figures 3B,C).

Furthermore, the Western blot results revealed that SHFKO effectively modulated the levels of antioxidant proteins (SOD-1, HO-1, catalase, Nrf2) in cutaneous tissue, thereby attenuating X-ray radiation-induced oxidative stress levels in the skin.

### 3.4 SHFKO promotes radiation-induced dermatitis recovery via the MAPK/MRK signaling pathway

**FIGURE 4:**
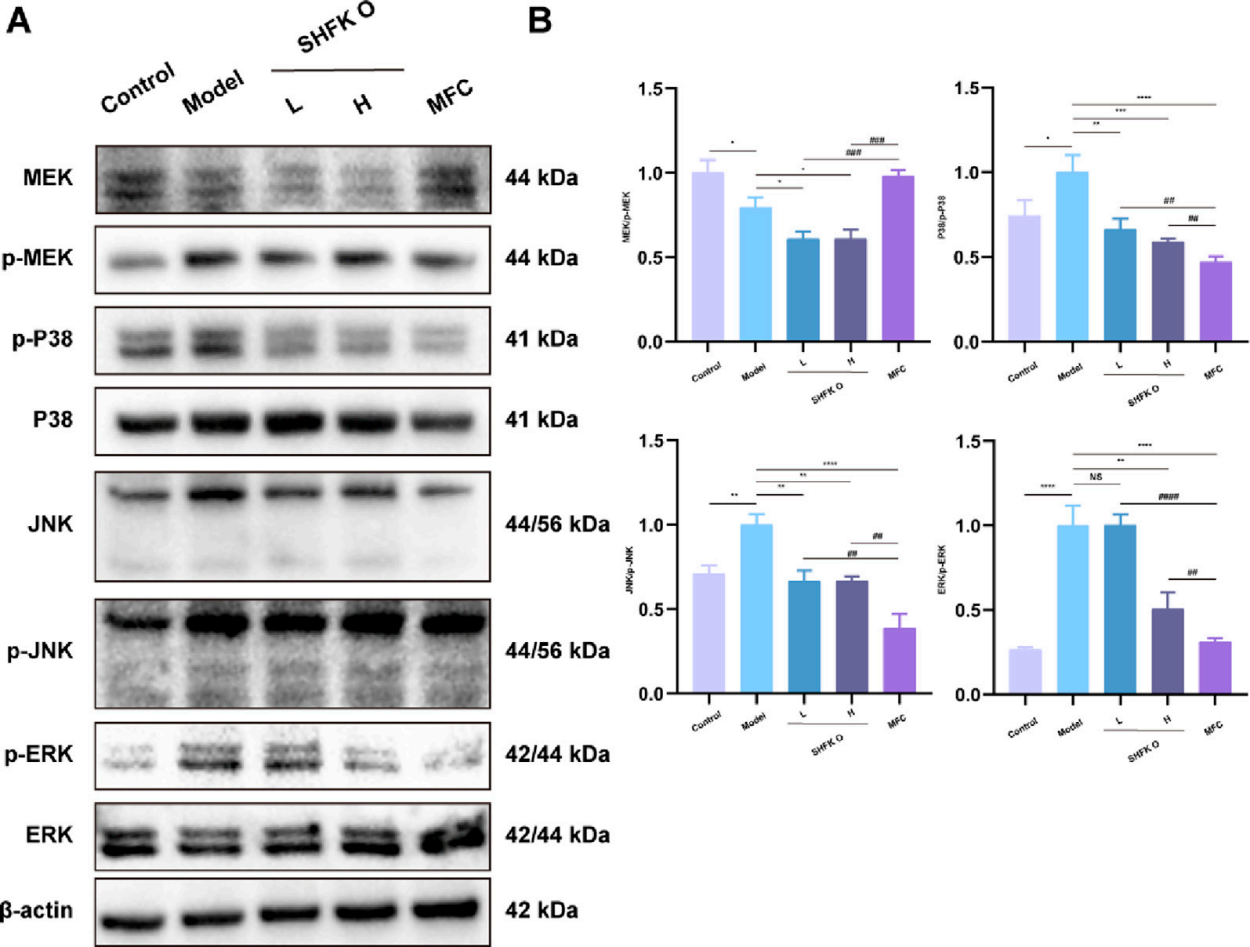
SHFKO promotes cutaneous repair by modulating the MAPK signaling pathway. **(A)** Western blot analysis of the relative phosphorylation levels of MAPK pathway proteins (MEK, P38, JNK, ERK) in cutaneous tissue specimens. **(B)** Quantitative densitometric analysis of relative protein levels presented as bar graphs. MFC vs. model and SHFKO vs. model: *p < 0.05, **p < 0.01, ***p < 0.001; SHFKO vs. MFC: #*p* < 0.05, ##*p* < 0.01, ###*p* < 0.001; NS, not significant (n = 8).

A Western blot analysis was used to evaluate the phosphorylation levels of classical MAPK signaling pathway proteins in cutaneous tissue, including MEK, P38, JNK, and ERK,. As shown in Figures 4A,B, following 4 weeks of therapeutic intervention, the model group exhibited elevated P38, JNK, and ERK protein levels compared to the control group, while MEK protein levels were diminished. SHFKO treatment significantly decreased MAPK/ERK pathway protein levels, with the high-concentration group demonstrating the highest inhibitory effects. Notably, while SHFKO showed slightly lower inhibition of most of the proteins compared to the MFC group, its regulatory effect on MEK protein levels was comparable.

The experimental findings revealed that the therapeutic mechanism of SHFKO primarily operated by inhibiting the MAPK/ERK pathway and regulating ROS levels. The MAPK/ERK signaling cascade is a classical inflammatory regulatory pathway, with JNK, P38, and ERK serving as pivotal mediators. Upregulation of these proteins typically induces keratinocytes within the epidermis to produce elevated levels of pro-inflammatory mediators, including IL-6, MMP13, and IL-1β.

Studies have demonstrated that radiation-induced ROS accumulation leads to enhanced levels of JNK, P38, and ERK in cutaneous tissue. Consequently, the ability of SHFKO to modulate the cross-talk between these two pathways underlies its enhanced therapeutic effects in ameliorating inflammation and promoting lesion repair.

### 3.5 SHFKO promotes recovery from radiation-induced dermatitis by modulating the NF-κB/PI3K-AKT signaling pathway

**FIGURE 5:**
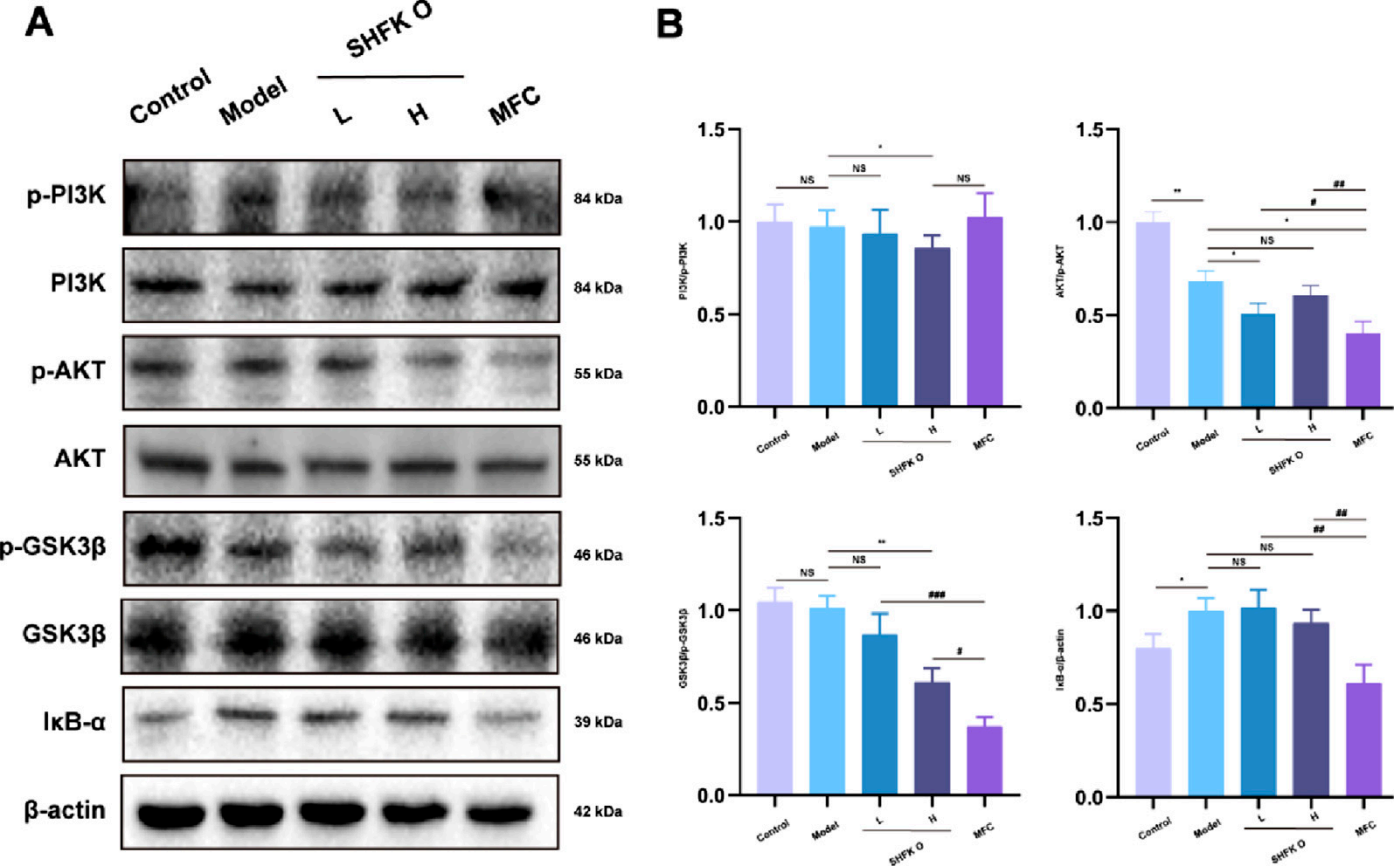
SHFKO mediates cutaneous repair by modulating the NF-κB/PI3K-AKT signaling pathway. **(A)** Western blot analysis of the relative phosphorylation levels of NF-κB/PI3K-AKT pathway proteins (PI3K, AKT, GSK3β, IκB-α) in cutaneous tissue. **(B)** Quantitative densitometric analysis of the relative protein levels presented as bar graphs. MFC vs. model and SHFKO vs. model: *p < 0.05, **p < 0.01, ***p < 0.001; SHFKO vs. MFC: #*p* < 0.05, ##*p* < 0.01, ###*p* < 0.001; NS, not significant (n = 8).

We further investigated the phosphorylation levels of proteins within the NF-κB/PI3K-AKT signaling pathway in the cutaneous tissue from each group, including the proteins PI3K, AKT, GSK3β, and IκB-α. As illustrated in Figures 5A,B, the high-concentration SHFKO group significantly inhibited PI3K phosphorylation, whereas the MFC group showed no apparent regulatory trend. The model group exhibited decreased levels of p-AKT compared to the control group, with the low-concentration SHFKO group demonstrating further suppression of p-AKT levels, while the high-concentration group showed no significant effect. Moreover, SHFKO demonstrated effects comparable to those of MFC in suppressing GSK3β phosphorylation levels, with both treatments exhibiting significant inhibitory effects. However, SHFKO showed no notable regulatory effects on IκB-α protein expression.

### 3.6 SHFKO enhances cutaneous wound healing quality

**FIGURE 6:**
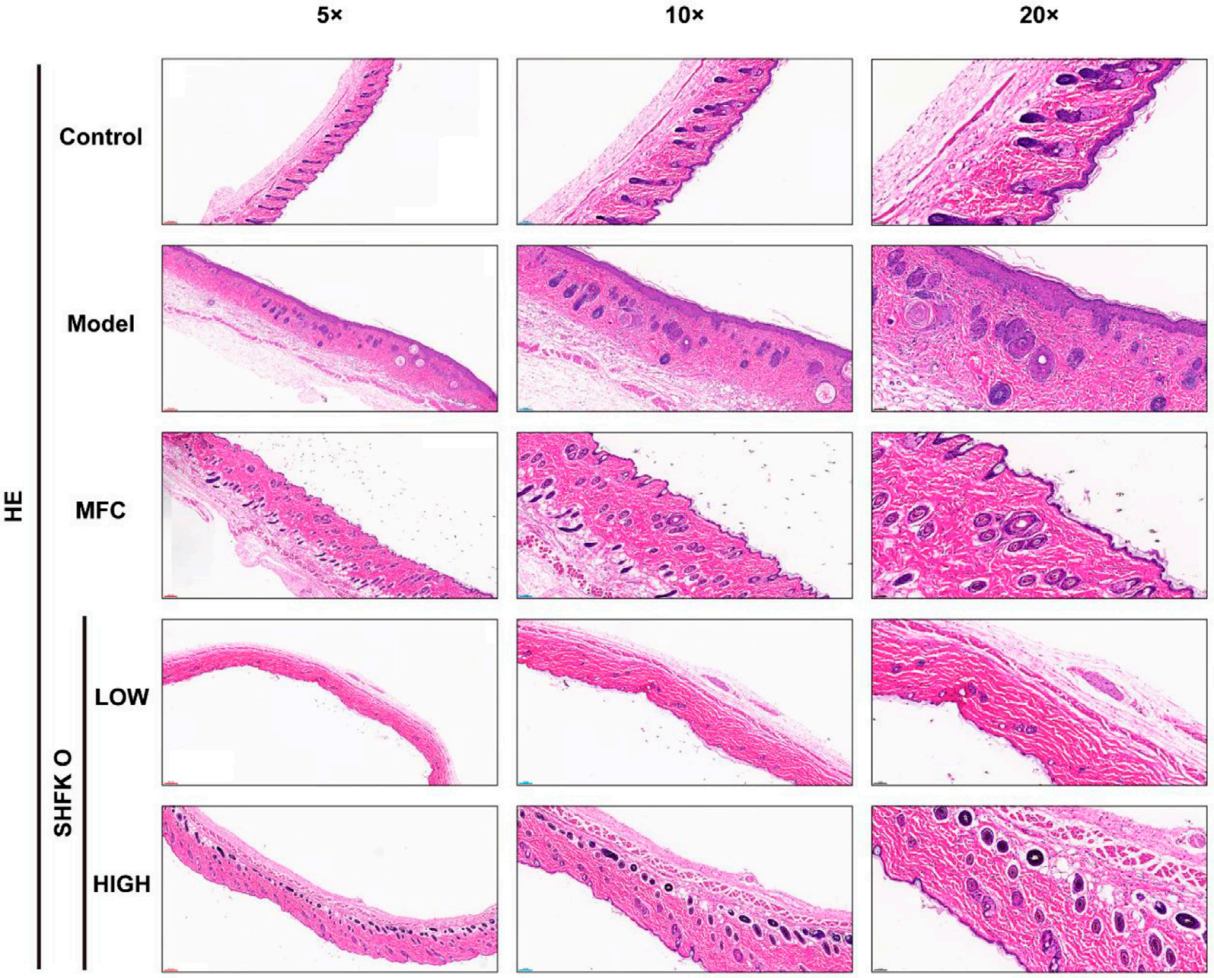
H&E-stained sections show that SHFKO enhances cutaneous repair quality and accelerates healing progression. Scale bars: 50 μm, 100 μm, and 200 μm.

Hematoxylin and eosin (H&E) staining was used to evaluate the ability of SHFKO to promote and enhance cutaneous wound healing quality. As shown in Figure 6, the control group exhibited a normal cutaneous architecture with distinct and intact stratification, characterized by a compact epidermis of uniform thickness, an absence of significant inflammatory cell infiltration, and well-preserved skin appendages with clear tissue organization.

In contrast, the model group demonstrated marked structural disorganization, with epidermal hyperplasia (predominantly in the stratum spinosum) and an irregular thickness, accompanied by hyperkeratosis. The dermis and hypodermis showed notable inflammatory cell infiltration. Hair follicles displayed irregular dimensions and appeared occluded, with reduced mature hair bulbs and an absence of distinct sebaceous glands.

The MFC (positive control) group exhibited a largely restored tissue architecture with distinct stratification. The epidermal thickness showed no significant hyperplasia, although some regional variations in thickness were observed. Notably, the dermis and hypodermis were devoid of inflammatory cell infiltration. Multiple normal hair follicles were present, with increased sebaceous glands compared to the model group.

In the low-dose SHFKO group, the tissue architecture demonstrated a basic structural organization with discernible stratification. While epidermal thickening was observed, some regions displayed thinning with insufficient keratinization. Minimal inflammatory cell infiltration was noted in the dermis and hypodermis. Skin appendages and sebaceous glands were sparse.

The high-dose SHFKO group demonstrated a well-organized tissue architecture with intact stratification. The epidermis recovered to a normal thickness, although some regional thinning persisted. Notably, the dermis and hypodermis showed no significant inflammatory cell infiltration. Skin appendages were abundant and uniformly sized, with multiple sebaceous glands and mature hair follicles present.

### 3.7 SHFKO reduces inflammatory mediators in cutaneous tissue

**FIGURE 7:**
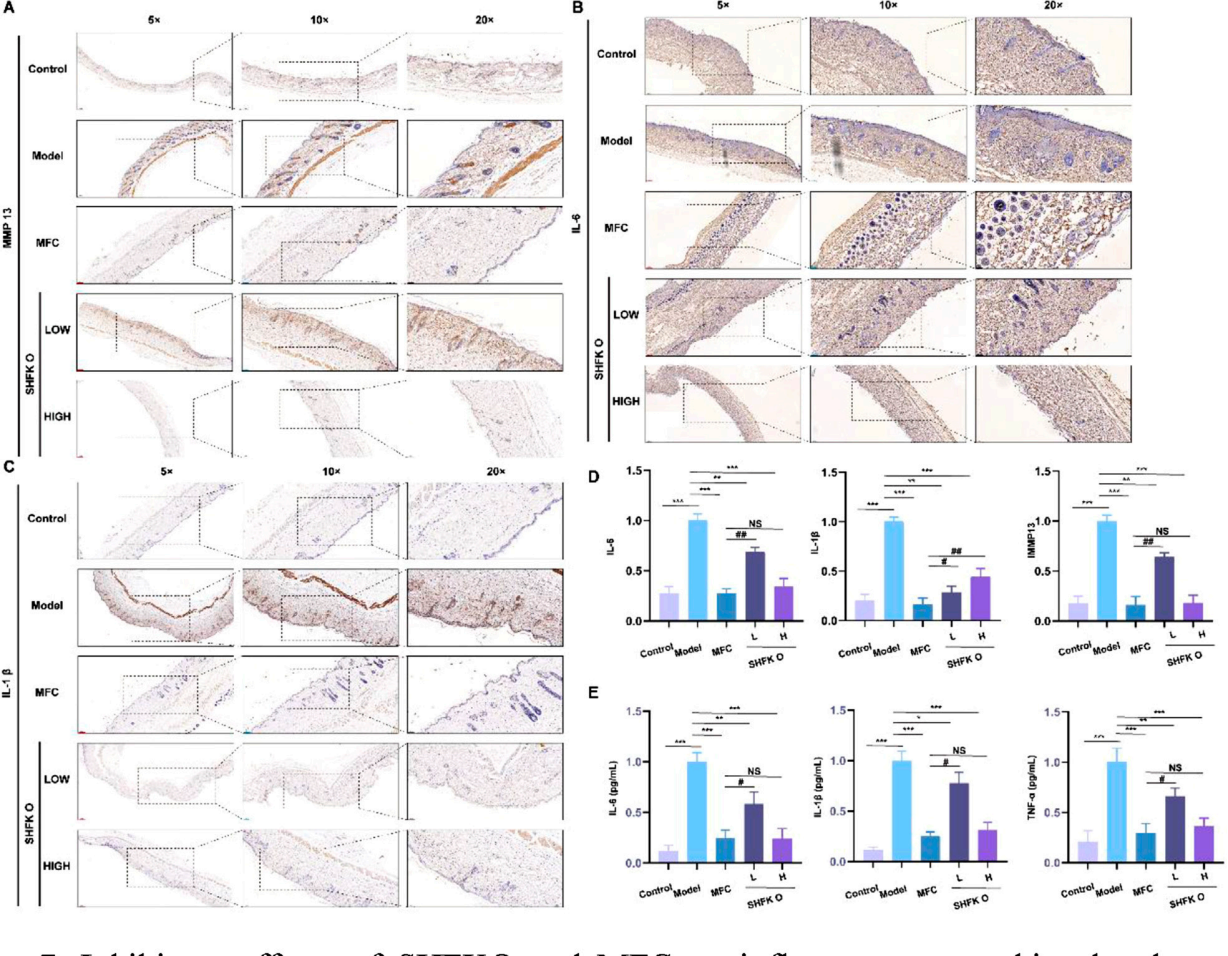
Inhibitory effects of SHFKO and MFC on inflammatory cytokine levels. **(A–D)** SHFKO and MFC exhibited congruent mechanisms in the attenuation of X-ray-induced local inflammatory cytokine levels (IL-1β, IL-6, and MMP13) within dermal tissue. **(E)** ELISA quantification demonstrated concentration-dependent inhibitory effects of both SHFKO and MFC on systemic inflammatory mediators (IL-1β, IL-6, and TNF-α) in serum, with marked attenuation observed at elevated therapeutic concentrations. MFC vs. model and SHFKO vs. model: *p < 0.05, **p < 0.01, ***p < 0.001; SHFKO vs. MFC: #*p* < 0.05, ##*p* < 0.01, ###*p* < 0.001; NS, not significant (n = 8).

Following X-ray irradiation, aberrant upregulation of IL-1β and IL-6 in cutaneous tissue orchestrates neutrophil infiltration through the selective activation of the NF-κB signaling pathways. This cascade potentiates ROS overproduction, thereby establishing a self-perpetuating pathological cycle intertwining inflammatory responses and oxidative stress (DOI: 10.7150/thno.97854). Comparative analyses using immunohistochemistry (Figures 7A–D) and ELISA (Figure 7E) demonstrated markedly elevated levels of IL-1β, IL-6, TNF-α, and MMP13 in both dermal tissue and serum of the model group. Notably, SHFKO and MFC treatment produced targeted suppression of these molecular mediators. Mechanistic investigations revealed that SHFKO mitigated MMP13-mediated extracellular matrix degradation by blocking the IL-1β/NF-κB signaling axis. Western blot analyses confirmed the ability of SHFKO to attenuate ROS generation, effectively disrupting this maladaptive feedback loop. The observed multi-targeted therapeutic modality of SHFKO demonstrated a striking congruence with the MFC mechanism of action, collectively suggesting their shared effects in reconfiguring the cutaneous reparative microenvironment through specific modulation of core inflammatory networks.

### 3.8 SHFKO mitigates radiation-induced skin apoptosis to accelerate wound repair

X-ray irradiation triggers localized inflammatory cascades and a ROS -antioxidant activity imbalance in dermal tissues, establishing a pro-oxidative microenvironment that perpetuates oxidative damage. This pathological state activates the caspase-9/3 apoptotic pathway, precipitating apoptotic cell death (Shi et al., 2022). To investigate the therapeutic mechanisms of SHFKO, we performed a Western blot quantification and immunohistochemical analysis of the apoptosis-related biomarkers cleaved-caspase-9, cleaved-caspase-3, and Bcl-2.

**FIGURE 8:**
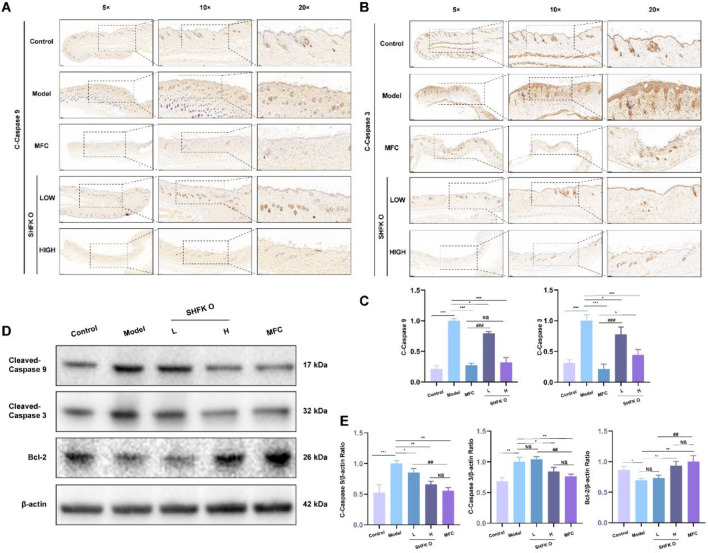
Pharmacological attenuation of radiation-induced apoptosis by SHFKO. **(A–C)** Histological and biochemical profiling demonstrated differential attenuation of apoptotic signaling across treatment groups, with SHFKO and MFC showing a dose-dependent decrease in caspase activation. **(D,E)** Western blot quantification confirmed the concentration-responsive suppression of pro-apoptotic mediators (cleaved-caspase-9/3) and increased Bcl-2 levels, with optimal SHFKO concentrations mirroring the therapeutic effects of MFC. MFC vs. model and SHFKO vs. model: *p < 0.05, **p < 0.01, ***p < 0.001; SHFKO vs. MFC: #*p* < 0.05, ##*p* < 0.01, ###*p* < 0.001; NS, not significant (n = 8).

As shown in Figures 8A–C, the model group specimens exhibited pronounced apoptotic activation post-irradiation, with cleaved-caspase-9 and cleaved-caspase-3 demonstrating significant upregulation compared to controls (*p* < 0.01). SHFKO and MFC treatments substantially attenuated levels of these apoptotic markers in a dose-responsive manner. Western blot analyses (Figures 8D,E) further revealed that higher SHFKO concentrations correlated with the progressive suppression of pro-apoptotic proteins (cleaved-caspase-9/3) and concomitant upregulation of anti-apoptotic Bcl-2, achieving pharmacodynamic convergence with MFC at higher dosages.

### 3.9 SHFKO modulates CXCL9 and integrin β1 to remodel the inflammatory response and promote skin damage repair

CXCL9, a macrophage-derived chemokine, performs dual roles in tissue injury through inflammatory regulation and pathogen clearance while simultaneously promoting fibroblast migration to enhance extracellular matrix remodeling (Kersh et al., 2024). Integrin β1 functions as a critical mediator of keratinocyte and fibroblast motility, driving critical repair processes that include re-epithelialization, granulation tissue formation, and coordinated cellular proliferation and migration (Shi et al., 2022). Mechanistic analyses have suggested that CXCL9 may accelerate wound closure by upregulating integrin β1 expression or potentiate its bioactivity, thereby synergistically enhancing keratinocyte and fibroblast migration (Wang J. et al., 2023).

**FIGURE 9:**
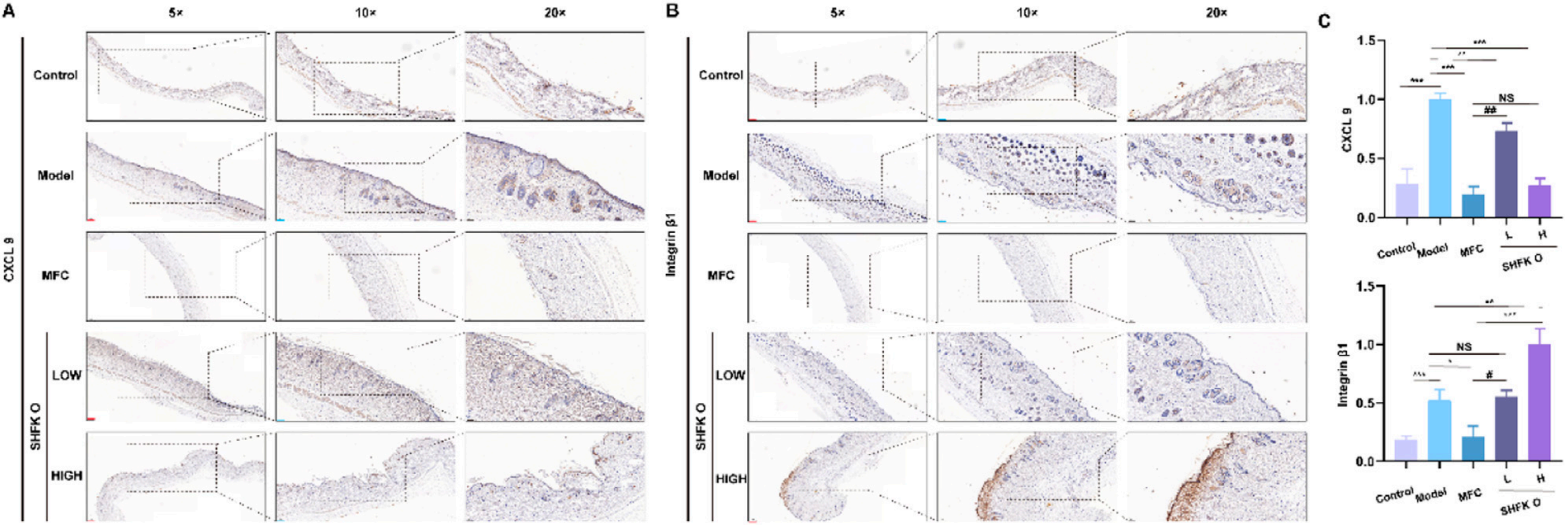
Pharmacological regulation of CXCL9 and integrin β1 by SHFKO facilitates dermal repair through inflammatory remodeling. **(A–C)** SHFKO and MFC orchestrated reciprocal modulation of CXCL9 and integrin β1 expression profiles, demonstrating a therapeutic ability to reconfigure localized inflammatory dynamics while enhancing tissue regeneration. MFC vs. model and SHFKO vs. model: *p < 0.05, **p < 0.01, ***p < 0.001; SHFKO vs. MFC: #*p* < 0.05, ##*p* < 0.01, ###*p* < 0.001; NS, not significant (n = 8).

Immunohistochemical profiling revealed dynamic changes in CXCL9 and integrin β1 levels with both SHFKO and MFC intervention (Figures 9A–C). Post-X-ray irradiation, model group specimens exhibited marked CXCL9 upregulation compared to controls (*p* < 0.01), indicating macrophage-driven CXCL9 overproduction during early reparative phases. Notably, SHFKO and MFC administration reversed this trend, suppressing CXCL9 expression in a dose-responsive manner. Conversely, integrin β1 demonstrated progressively increased levels correlating with wound maturation stages, with the highest levels at high SHFKO concentrations—a pattern that contrasted sharply with the CXCL9 dynamics.

## 4 Discussion

The pathophysiological mechanisms underlying ARD include stem and progenitor cell depletion within the basal and dermal layers, and fibrotic progression promoted by inflammatory mediators through multicellular interactions (Yarnold and Brotons, 2010; Fijardo et al., 2024). Ionizing radiation induces DNA double-strand breaks through both direct and indirect mechanisms, resulting in immediate cellular damage and cascading effects on cutaneous tissue (Liu and Wang, 2021). This damage compromises the proliferative capacity of stem and progenitor cells in the basal layer, suppresses the survival of epidermal keratinocytes, and impedes the synthesis and release of growth factors (Rosenthal et al., 2019; Liu et al., 2017; Liu et al., 2022). These processes collectively accelerate keratinocyte senescence and apoptosis and inhibit endothelial cell and fibroblast proliferation, consequently impairing angiogenesis and diminishing collagen synthesis, thereby prolonging the tissue repair process (Piao and Boeravinone, 2023; Karbasforooshan et al., 2019).

Pattern recognition receptors expressed by keratinocytes have the capacity to recognize both pathogen-associated molecular patterns and damage-associated molecular patterns (Tewary et al., 2024). Radiation exposure induces signal transduction between keratinocytes and immune cells through pro-inflammatory mediators, activating pivotal pathways, including PI3K/AKT, MAPK, and NF-κB, which subsequently trigger second messenger system activation, pro-inflammatory gene transcription, and cytokine cascade responses (Müller and Meineke, 2007). These molecular events facilitate the transendothelial migration of immune cells from the peripheral circulation to irradiated cutaneous tissue (Tewary et al., 2024; Müller and Meineke, 2007; Harjunpää et al., 2019). Matrix metalloproteinase activation results in the degradation of dermal matrix components and basement membranes, while upregulated expression of endothelial adhesion molecules further potentiates immune cell infiltration (Müller and Meineke, 2007; Rübe et al., 2024; Kim et al., 2013). These molecular events represent a characteristic signature of radiation-induced cutaneous injury and are cardinal indicators of therapeutic radiation-mediated tissue damage (Ryan, 2012; Müller and Meineke, 2007; Harjunpää et al., 2019; Rübe et al., 2024; Kim et al., 2013; Amber et al., 2014).

TCM formulations have been explored for potential therapeutic applications in the clinical management of ARD (Yu et al., 2022). Previous studies suggest that botanical drug preparations may exert anti-inflammatory and pro-reparative effects by modulating multiple signal transduction pathways (Yu et al., 2022). The present study used mass spectrometry analysis to characterize the metabolites of SHFKO and evaluated its pharmacological effects through both *in vitro* radiation injury models and *in vivo* ARD murine models. Our findings indicated that SHFKO may ameliorate the pathological progression of ARD in our experimental models by augmenting the antioxidant defense system and modulating inflammatory responses, with the observed changes associated with the suppression of PI3K/AKT pathway activation and the promotion of MAPK/ERK phosphorylation.

The ROS family encompasses the superoxide anion (O2·-), hydrogen peroxide (H_2_O_2_), hydroxyl radical (·OH), and singlet oxygen (1O2) molecules that exhibit heightened chemical reactivity due to the presence of unpaired electrons in their outer electron configurations (Nakai and Tsuruta, 2021). Under physiological conditions, the endogenous antioxidant defense system, comprising superoxide dismutase, catalase, and glutathione peroxidase, maintains redox homeostasis (Kim et al., 2013). Ionizing radiation initiates the radiolysis of intracellular water molecules, while leukocytes and fibroblasts, upon activation and infiltration in response to pro-inflammatory cytokine stimulation, generate supraphysiological levels of ROS, which catalyze lipid peroxidation and promote pro-inflammatory mediator release (Nakai and Tsuruta, 2021; Zhang et al., 2017). When the oxidative burden overwhelms the endogenous antioxidant defense capacity, protein conformational changes and DNA damage ensue, ultimately activating programmed cell death pathways (Nakai and Tsuruta, 2021; Zhang et al., 2017; Akay et al., 2004).

The present investigation observed that SHFKO treatment was associated with two antioxidant-related changes: decreased ROS generation and increased expression of SOD-1, HO-1, catalase, and NRF2. Mass spectrometry analysis identified multiple bioactive metabolites, including phellodendrine, previously reported to be associated with reduced ROS production; dehydrocorydaline, reported to influence mitochondrial membrane potential; and berberine, documented to interact with the NRF2/ARE pathway (Hu et al., 2022; Shi et al., 2025; Liu et al., 2024; Li et al., 2025; Chen et al., 2025; Nguyen et al., 2022; Wang et al., 2021). These components may contribute to the observed antioxidant effects through multiple mechanisms.

Macrophage polarization is pivotal in the pathological progression of ARD (Pasparakis et al., 2014). Radiation induces macrophage polarization toward the pro-inflammatory M1 phenotype, resulting in the release of pro-inflammatory cytokines, including IL-6 and IL-1β (Xia et al., 2023). M2-type macrophages produce anti-inflammatory mediators, such as IL-10, thereby performing immunomodulatory functions (Xia et al., 2023). Disruption of immune homeostasis can lead to excessive M1 phenotype polarization, promoting immune cell recruitment and potentially precipitating chronic inflammation (Xia et al., 2023; Shi and Shiao, 2018; Hassanshahi et al., 2022). IL-6, an early inflammatory biomarker, induces tissue-resident cells to produce pro-inflammatory cytokines (Xia et al., 2023; Shi and Shiao, 2018). Studies have confirmed that anti-IL-6 monoclonal antibodies can mitigate radiation-induced adverse effects (Marmary et al., 2016; Tartakovsky et al., 1993; Fedorocko et al., 2002; Chen et al., 2001; Meirovitz et al., 2010). TNF-α upregulates MMP expression, accelerating matrix degradation and cutaneous senescence (Hwang et al., 2012; Herouy et al., 2001). Interleukin-1 (IL-1), also referred to as lymphocyte-stimulating factor, is a central mediator of apoptosis and sterile inflammation triggered by numerous stimuli (Janko et al., 2012).

The potential anti-inflammatory mechanisms of SHFKO may be related to multiple bioactive components identified in our analysis: phellodendrine has been associated with reduced IL-6 and TNF-α transcription in studies involving the IL-17/NF-κB signaling axis; dehydrocorydaline has been linked to changes in the PI3K/Akt pathway and shifts toward M2 macrophage polarization in previous reports; berberine has been reported to interfere with NLRP3 inflammasome assembly in isolated systems; and baicalein and coptisine have been associated with MMP-13 downregulation in separate studies (Liu et al., 2010; Xu et al., 2022). However, it is important to note that these mechanisms were identified in different experimental contexts, and their relevance to the observed effects of the complete SHFKO formulation in our ARD models requires validation through targeted mechanistic studies. The multi-component nature of TCM formulations presents both opportunities and challenges for understanding their therapeutic mechanisms, highlighting the need for systematic approaches to dissect component-specific contributions.

During the inflammatory response, interferon-γ (IFN-γ) is released by cells participating in both innate and adaptive immunity, including neutrophils, tissue macrophages, natural killer cells, and Th1 lymphocytes (Richmond et al., 2023). IFN-γ induces neutrophil chemotaxis toward inflammatory sites and binds to type I and II receptors on the surface of tissue macrophages, activating macrophages and promoting their secretion of the chemokine CXCL9 (Richmond et al., 2023). In our study, SHFKO treatment was associated with reduced CXCL9 levels in the cutaneous tissue of ARD murine models, though the direct causal relationship requires further validation. CXCL9, a chemokine with cytotoxic and pro-apoptotic properties, facilitates Th1 and T cell recruitment and activation by engaging with the CXCR3 receptor, participates in immune and inflammatory response pathways, and has been implicated in regulating vascular remodeling by interfering with vascular endothelial growth factor signaling, potentially affecting angiogenesis (Yang et al., 2020; Chen et al., 2022).

Integrin β1, a transmembrane heterodimer formed by the non-covalent association of α and β subunits, is involved in cell adhesion, leukocyte recruitment regulation, and wound repair processes (Wang et al., 2020). As a mediator of cell-cell and cell-extracellular matrix interactions, integrin β1 contributes to maintaining cutaneous function and promoting wound healing (Grose et al., 2002; Raghavan et al., 2000). Studies have demonstrated that the conditional knockout of integrin β1 in skin causes impaired epidermal proliferation, basement membrane formation failure, hemidesmosome instability, aberrant follicular development, and severe cutaneous blistering and hair defects (Raghavan et al., 2000). Integrin β1 deficiency in keratinocytes compromises cellular adhesion, impairs proliferation and migration, disrupts the epithelial architecture, and causes wound healing deficits, while its absence in fibroblasts induces dermal thinning and diminishes collagen expression (Grose et al., 2002). In murine wound models, integrin β1 expression was shown to be elevated at wound margins and then migrated toward the wound center, where it was associated with activation of the PI3K/Akt pathway, which correlated with reduced local inflammation and enhanced wound healing and tissue regeneration (Liu and Leask, 2013). In our study, SHFKO treatment was associated with increased integrin β1 expression, suggesting a potential mechanism for the observed tissue repair effects, though direct causation was not established.

The NF-κB, PI3K/Akt, and MAPK signaling pathways are important intracellular networks involved in the pathogenesis and progression of radiation dermatitis (Shen et al., 2022). The NF-κB signaling pathway is involved in pro-inflammatory molecule regulation (TNF-α, IL-1β, IL-6), immune responses, cellular proliferation, and apoptosis in both physiological and pathological contexts. In response to oxidative stress and ionizing radiation, DNA damage can lead to activation of NF-κB through p53, potentially inducing pro-apoptotic responses; during inflammation, lipopolysaccharide binding to TLR receptors on macrophage membranes may activate NF-κB, which can facilitate p65/p50 nuclear translocation, potentially promoting M1 macrophage polarization, and contributing to inflammatory responses (Ryan et al., 2000).

The PI3K/AKT signaling pathway participates in regulating cellular proliferation, differentiation, migration, angiogenesis, and metabolic processes, and is essential for contributes to maintaining cutaneous homeostasis. Activation of this pathway has been associated with the epithelial-mesenchymal transition, cutaneous wound healing, and follicular regeneration and may protect melanocytes from oxidative stress (Roy et al., 2023). Different AKT isoforms appear to have different regulatory effects on macrophage polarization, with PI3K/AKT potentially suppressing M1 polarization while favoring the M2 transition, which may contribute to anti-inflammatory processes. The MAPK signaling pathway comprises three major branches: ERK, JNK, and p38 MAPK. The ERK pathway primarily mediates cellular responses to stress stimuli and inflammatory mediators, while the JNK and p38 MAPK pathways are involved in inflammatory responses, stress, and apoptosis (Chang et al., 2019; Jiang et al., 2020; Chen et al., 2017; Yin et al., 2013; Stone et al., 2016; Xiao et al., 2017; Wang et al., 2017; Denat et al., 2014; Yang et al., 2016; Zhou et al., 2016). Our findings showed that SHFKO treatment was associated with alterations in these pathways, particularly suppression of PI3K/AKT and activation of MAPK/ERK, though the functional significance of these changes in mediating the observed therapeutic effects requires further investigation.

Numerous natural products and TCM formulations have been investigated for potential therapeutic applications acting through these signaling pathway regulatory mechanisms (Wang et al., 2024). Grape seed proanthocyanidins were reported to exhibit photoprotective effects associated with reduced NF-κB/p65 activation and nuclear translocation, and a modified Simiao Yong’an formula showed beneficial effects on radiation dermatitis with changes in MAPK/ERK phosphorylation, reduced activity of the PI3K/AKT pathway, and decreased levels of α-SMA, Colla2, and TGF-β1 expression (Yong et al., 2009; Xiong et al., 2021; Park et al., 2020; Wang Y. et al., 2023). These investigations suggest complex cross-regulatory relationships among NF-κB, PI3K/AKT, and MAPK signaling pathways, which may inform multi-target intervention approaches in radiation dermatitis.

The bioactive metabolites identified in SHFKO may contribute to the observed anti-inflammatory effects through multiple mechanisms. Previous studies have reported that berberine can influence integrin β1 phosphorylation and NF-κB activation; dehydrocorydaline has been associated with reduced inflammatory mediator release and PI3K/AKT pathway modulation; and coptisine may affect CXCL9 levels and MAPK/ERK signaling (Zeng et al., 2024; Zhang et al., 2024). In our study, SHFKO treatment was associated with changes in these pathways, including reduced NF-κB and PI3K/AKT activation, increased MAPK/ERK phosphorylation, and elevated integrin β1 expression. However, whether these individual components directly contribute to the observed effects when present together in the formulation remains to be determined. The relationship between these molecular changes and the therapeutic effects observed in our ARD models requires further investigation using component isolation and pathway-specific interventions.

This study provides important preclinical evidence supporting the therapeutic potential of SHFKO for radiation dermatitis; however, several significant limitations must be considered when interpreting the results. First, the murine model employed may not fully recapitulate human radiation dermatitis pathophysiology due to inherent differences in skin thickness, hair follicle density, immune responses, and healing mechanisms between mice and humans. Moreover, the single high-dose radiation protocol utilized may not accurately reflect the fractionated radiotherapy regimens commonly employed in clinical practice. Second, this study evaluated only two dose levels, precluding the establishment of a comprehensive dose-response relationship. Furthermore, the optimal therapeutic window, potential toxicity thresholds, stability, bioavailability, and pharmacokinetic profiles of individual components within the complex formulation remain uncharacterized. Finally, future research should address these limitations through systematic dose-response studies, bioavailability and pharmacokinetic analyses, component fractionation studies, mechanistic investigations employing pathway-specific interventions, comparative effectiveness studies with standard treatments, investigation of sex-specific responses and long-term outcomes, and ultimately, well-designed clinical trials to evaluate the safety and efficacy in patients with radiation dermatitis. Additionally, future studies could explore how Traditional Chinese Medicine theoretical frameworks might guide hypothesis generation for modern pharmacological research, while maintaining a clear distinction between traditional concepts and molecular mechanisms.

## 5 Conclusion

This preclinical study investigated the effects of SHFKO, a traditional Chinese medicine formulation, on radiation-induced skin injury using both *in vitro* and *in vivo* experimental models. Treatment with SHFKO was associated with reduced severity of radiation dermatitis in mice, as evidenced by improved histological scores, decreased inflammatory infiltration, and enhanced epithelial integrity. At the molecular level, SHFKO treatment correlated with increased expression of antioxidant enzymes (SOD-1, HO-1, catalase, and NRF2), reduced levels of pro-inflammatory cytokines (IL-6, IL-1β, TNF-α), and alterations in key signaling pathways, including suppression of PI3K/AKT and NF-κB activation alongside increased MAPK/ERK phosphorylation. Mass spectrometry analysis identified multiple bioactive compounds in SHFKO, including berberine, phellodendrine, and dehydrocorydaline, which have been previously reported to possess anti-inflammatory and antioxidant properties, though their specific contributions to the observed effects in our compound formulation remain to be determined.

While these findings suggest that SHFKO may have therapeutic potential for radiation dermatitis, several important caveats must be acknowledged. The observed associations between SHFKO treatment and molecular changes do not establish direct causal relationships, and the interactions between multiple components were not quantitatively assessed. The murine model used may not fully reflect human pathophysiology, and the translational relevance of these findings requires validation through clinical studies. Nevertheless, this study provides a foundation for further investigation of SHFKO as a potential therapeutic option for radiation dermatitis and demonstrates the value of systematically investigating traditional medicine formulations using modern pharmacological approaches. Future research should focus on dose optimization, mechanistic validation, component-specific contribution analysis, and ultimately, well-designed clinical trials to evaluate the safety and efficacy of SHFKO in patients receiving radiotherapy.

## Data Availability

The raw data supporting the conclusions of this article will be made available by the authors, upon reasonable request.
