# Supplementary material for: Sanhuang Fukang oil alleviates X-ray-induced skin injury by reducing inflammation and apoptosis: an in vivo study
Source: Front Pharmacol. 2026 Jan 6;16:1684426. doi: 10.3389/fphar.2025.1684426 (PMC12816242; doi:10.3389/fphar.2025.1684426)

MMP13

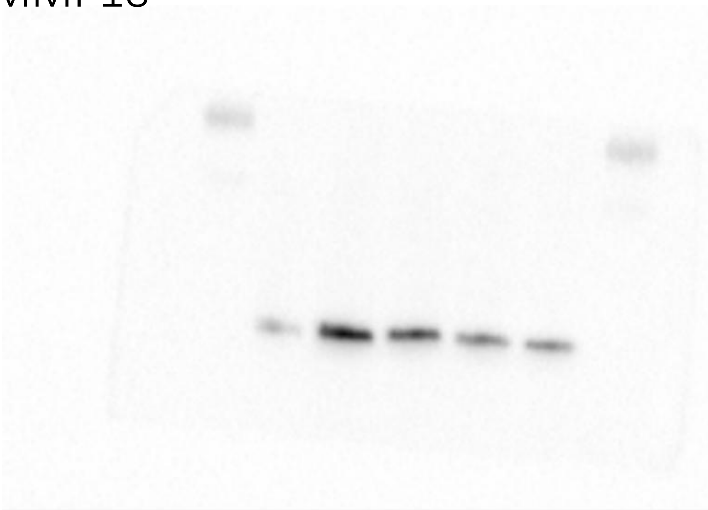

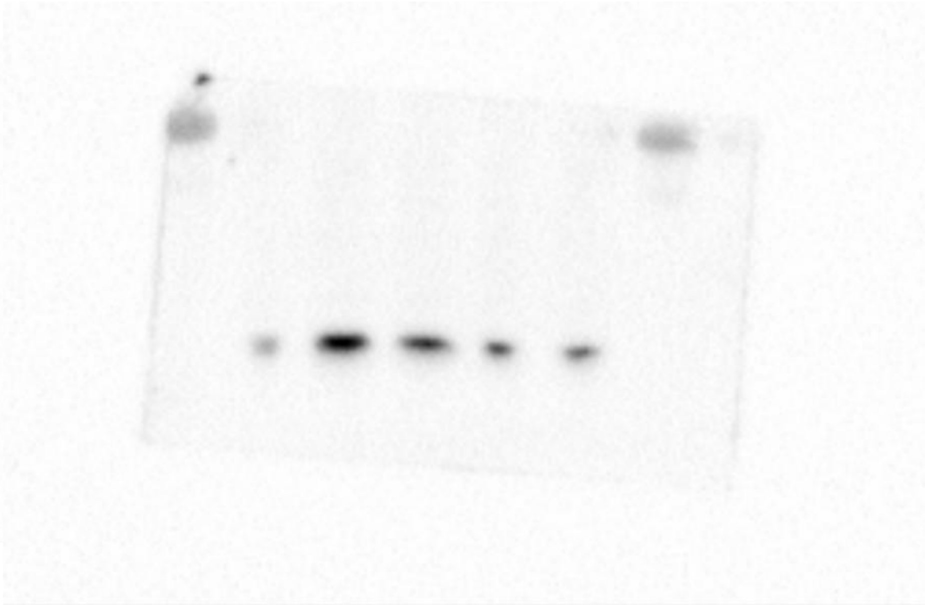

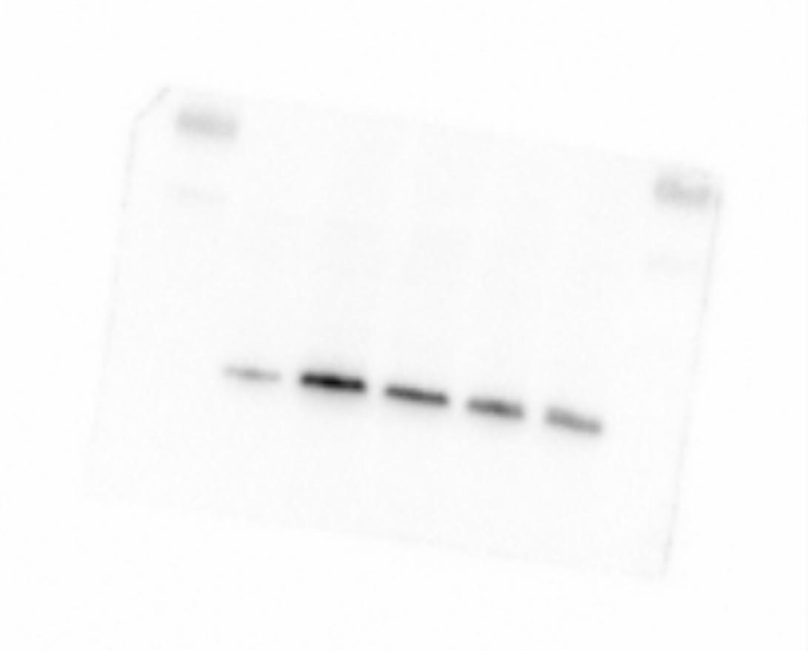

IL-1b

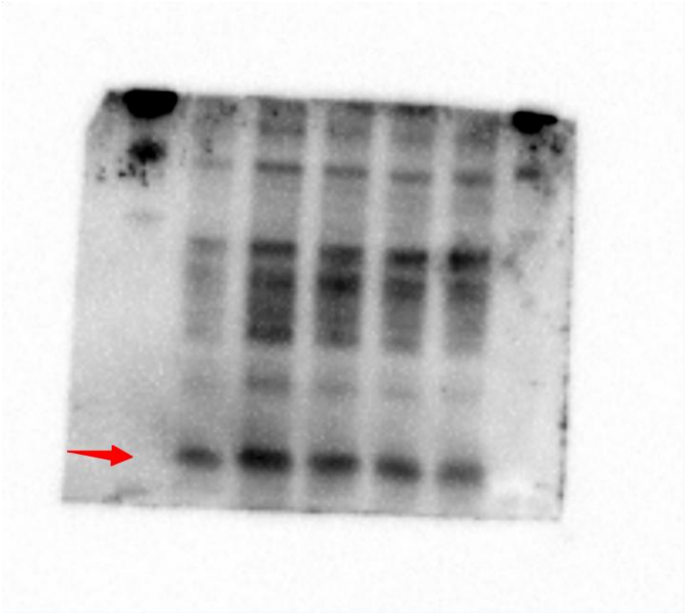

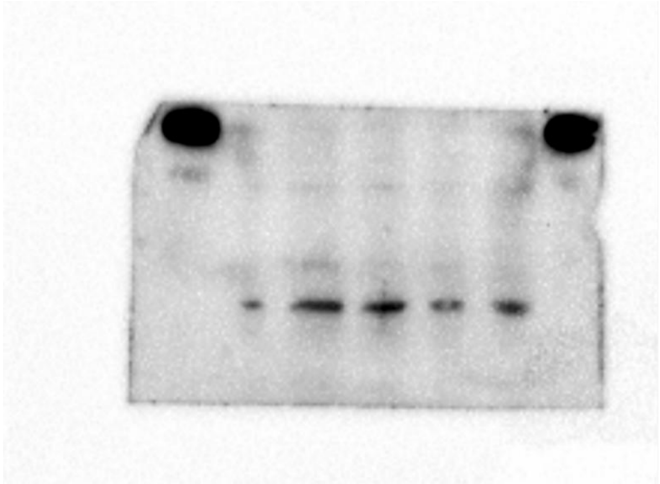

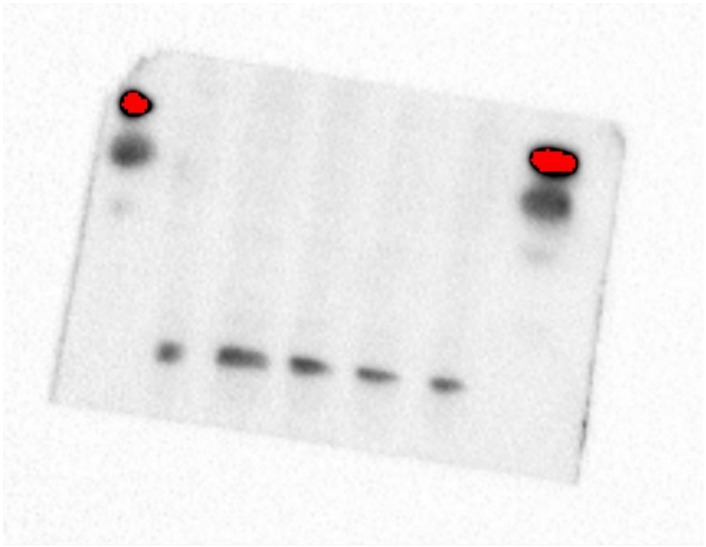

PPAR

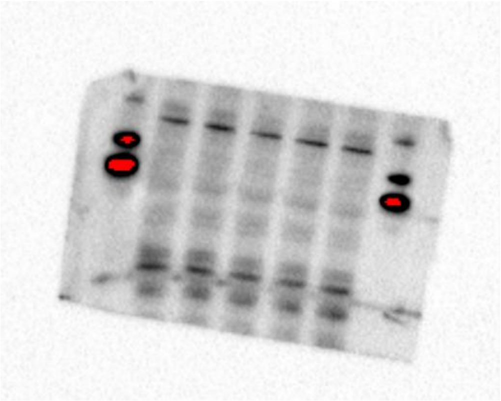

P-PPAR

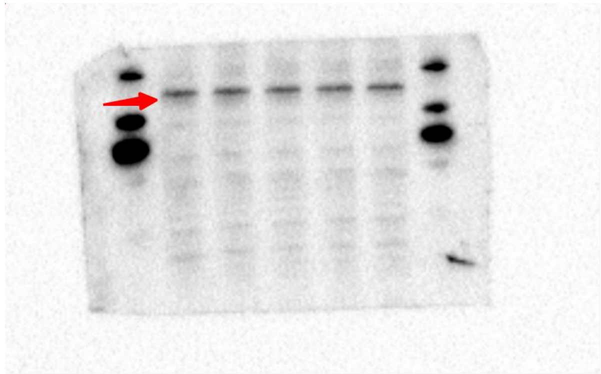

P-PPAR

PPAR

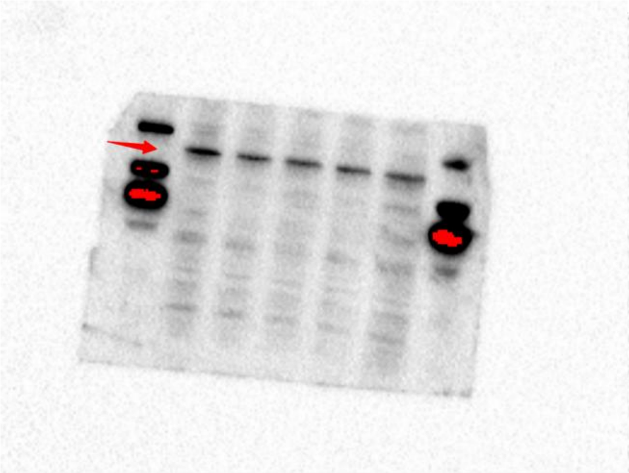

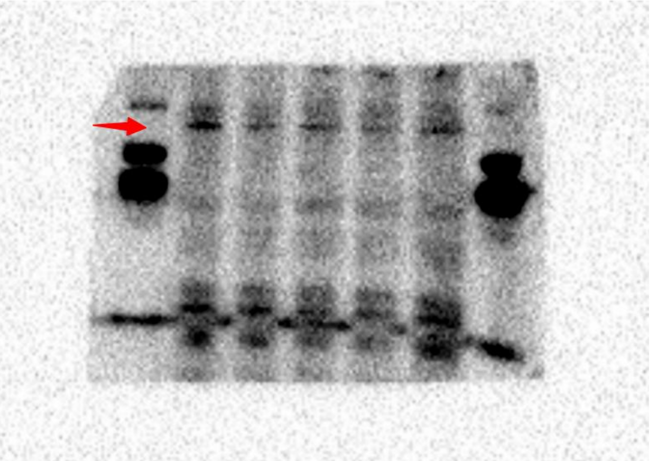

MEK

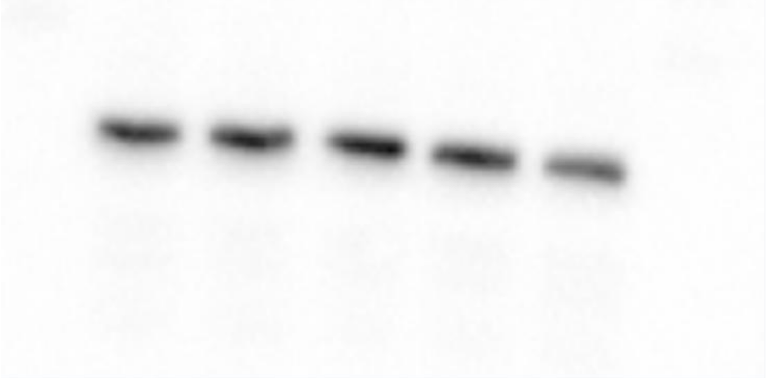

P-MEK

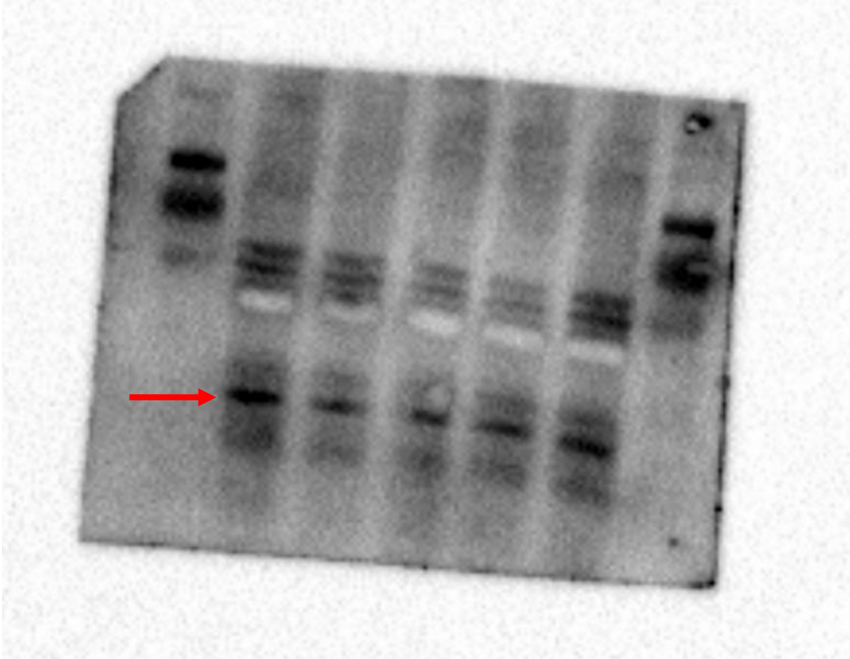

MEK

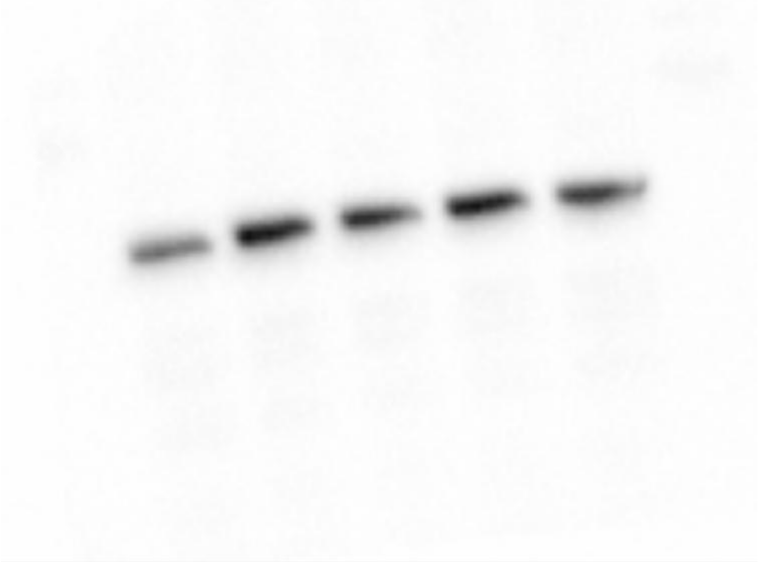

P-MEK

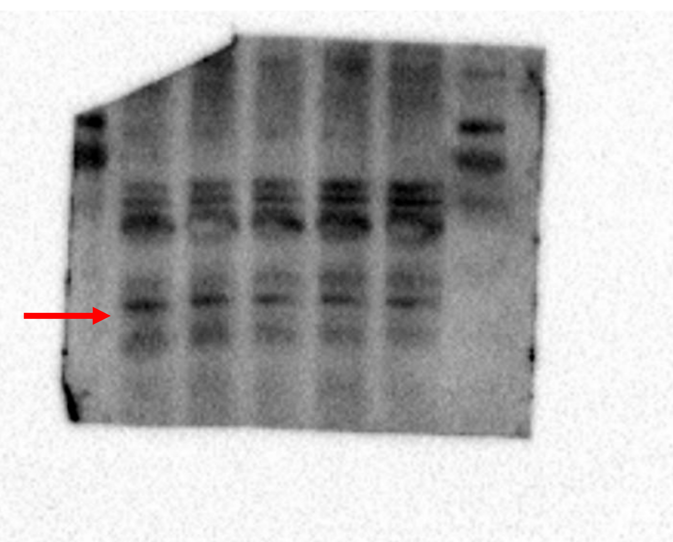

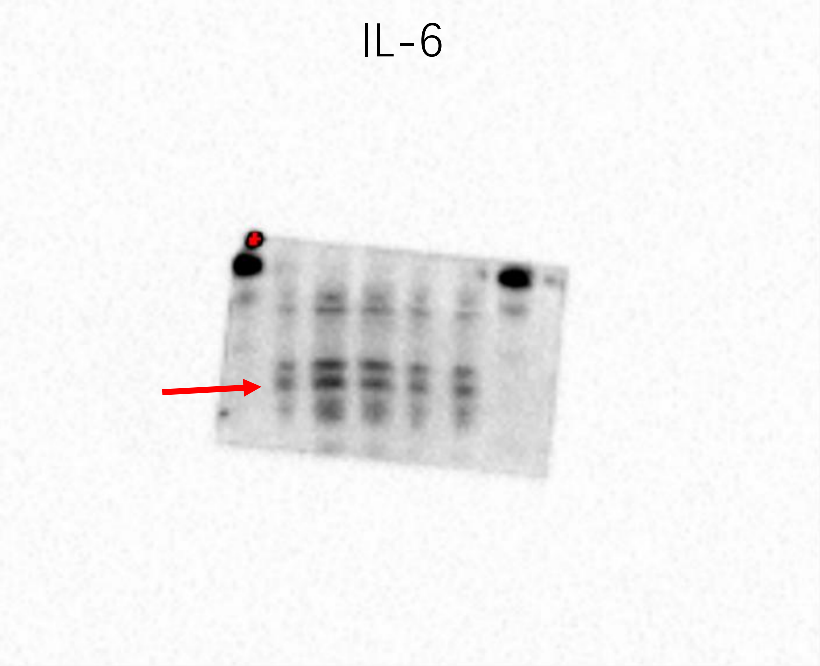
IL-6
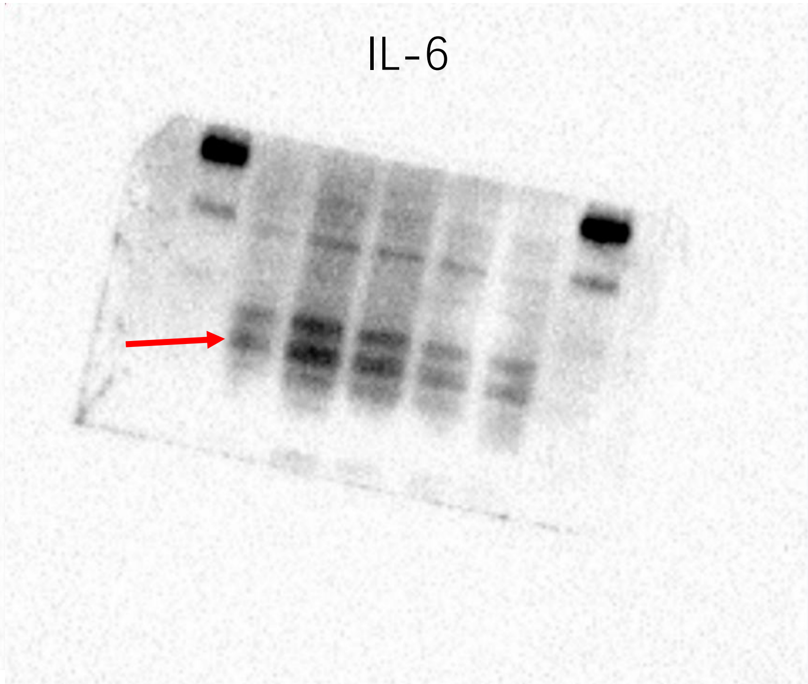
IL-6
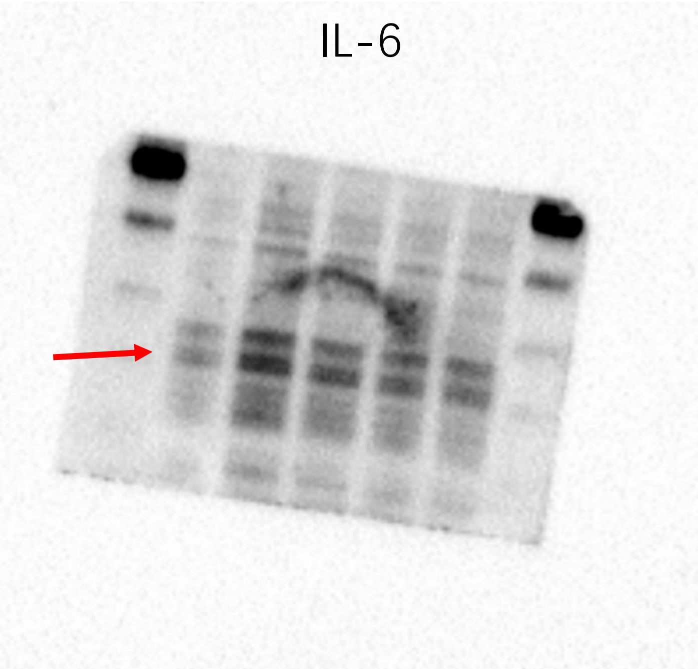
IL-6
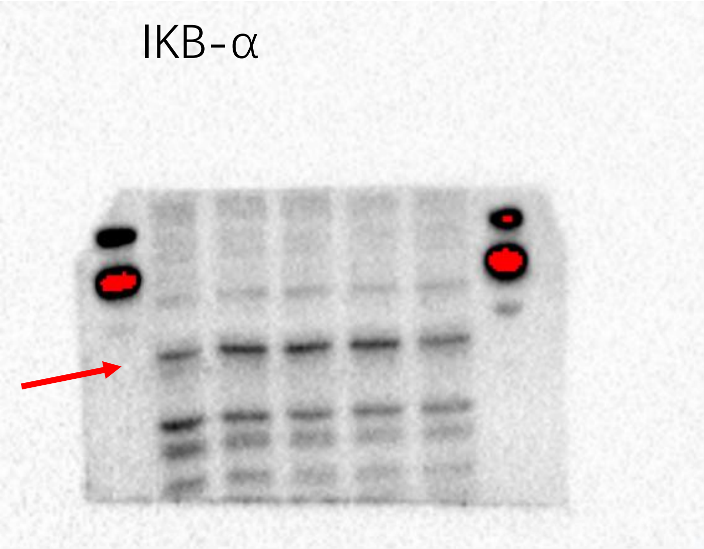
IKB-α
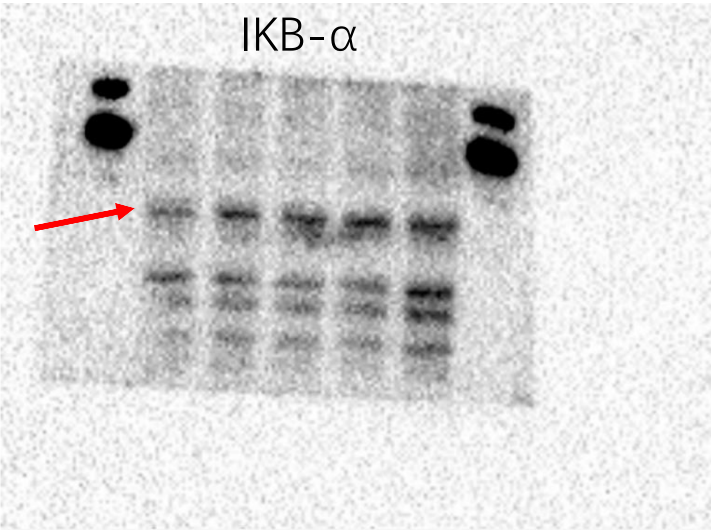
IKB-α

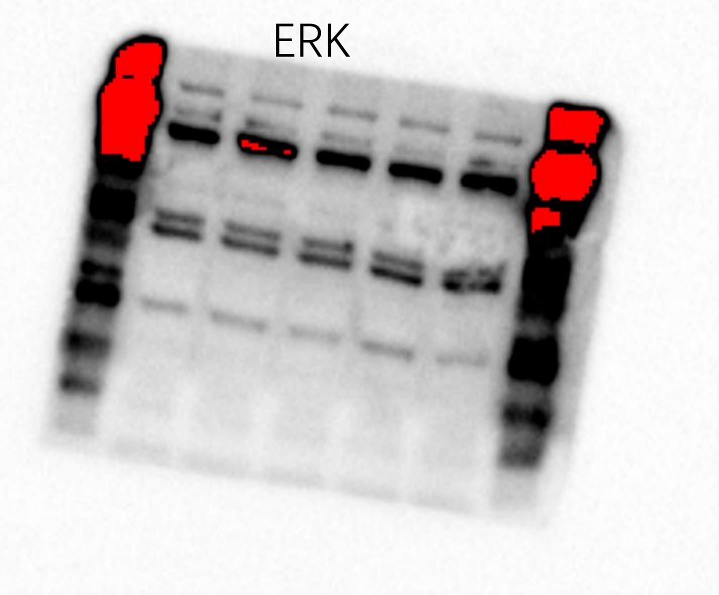
ERK

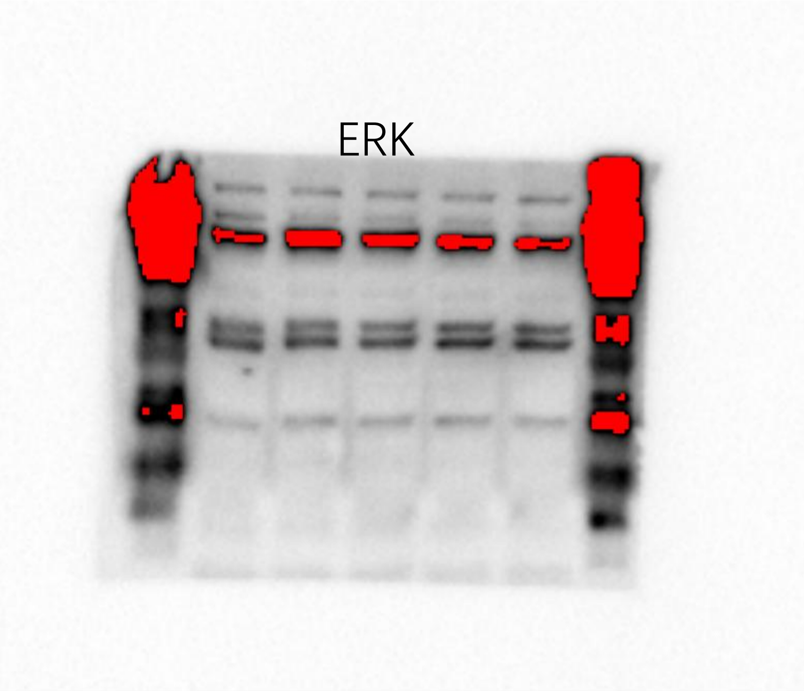
ERK

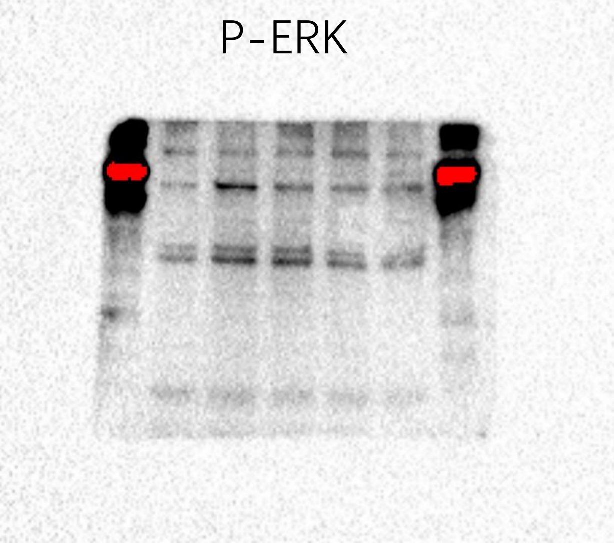
P-ERK

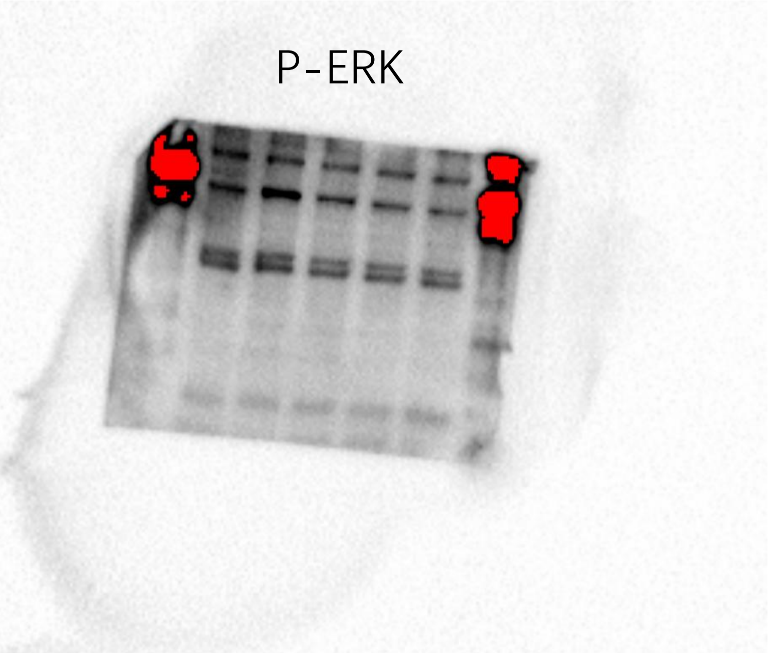
P-ERK

AKT

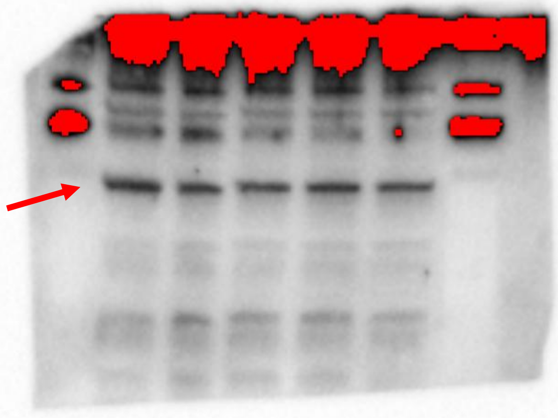

P-AKT

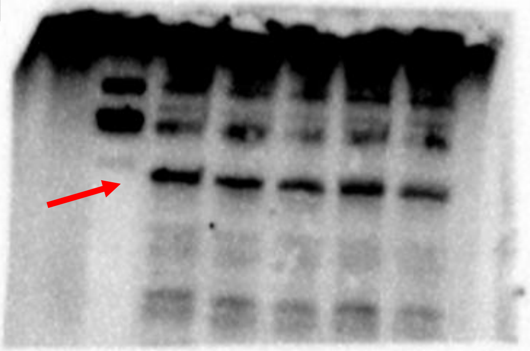

AKT

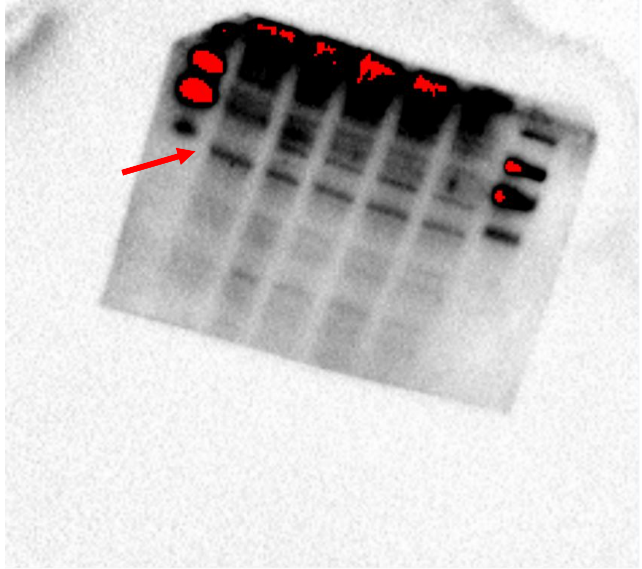

P-AKT

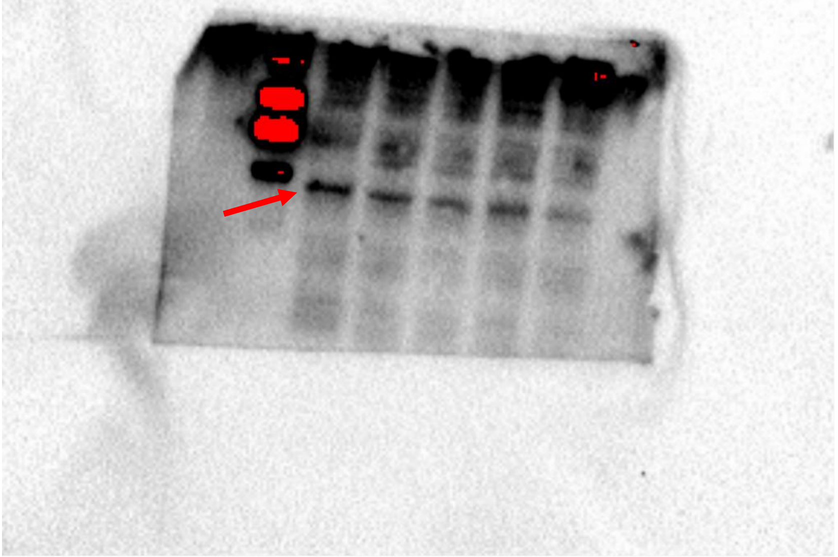

P38

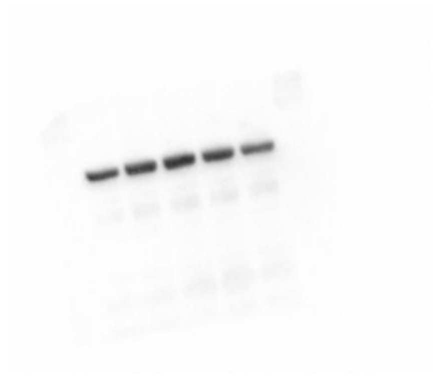

P38

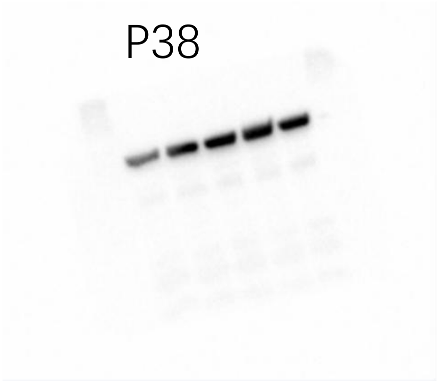

P-P38

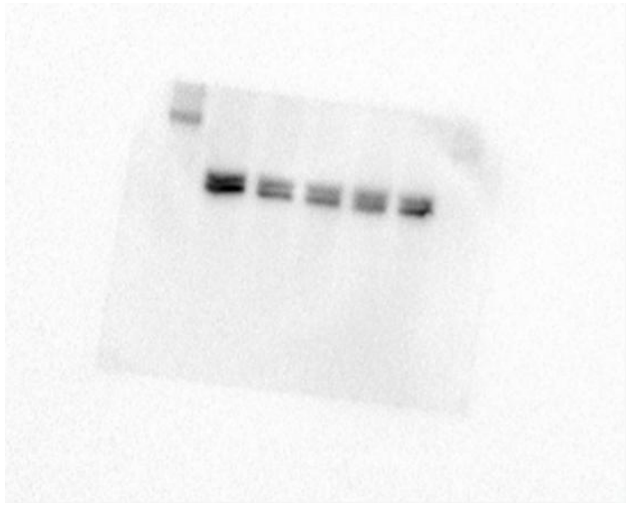

P-P38

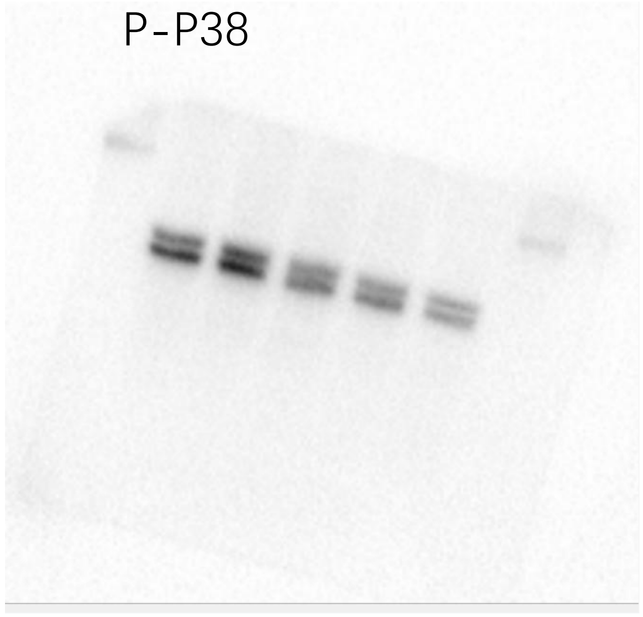

JNK

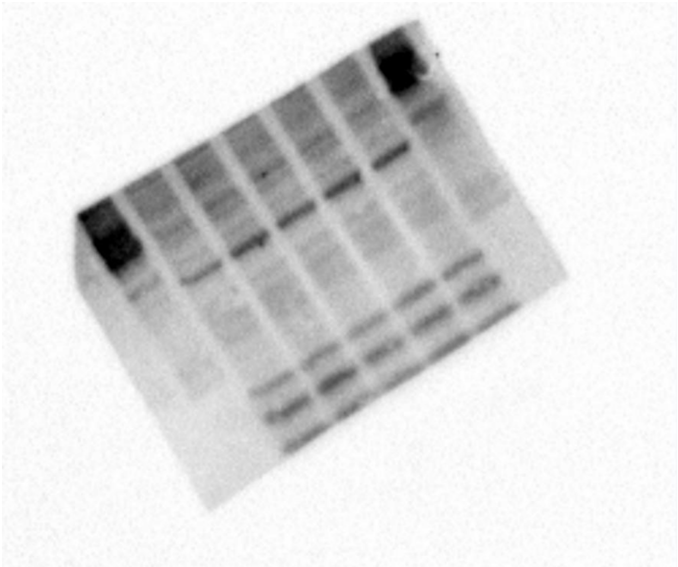

P-JNK

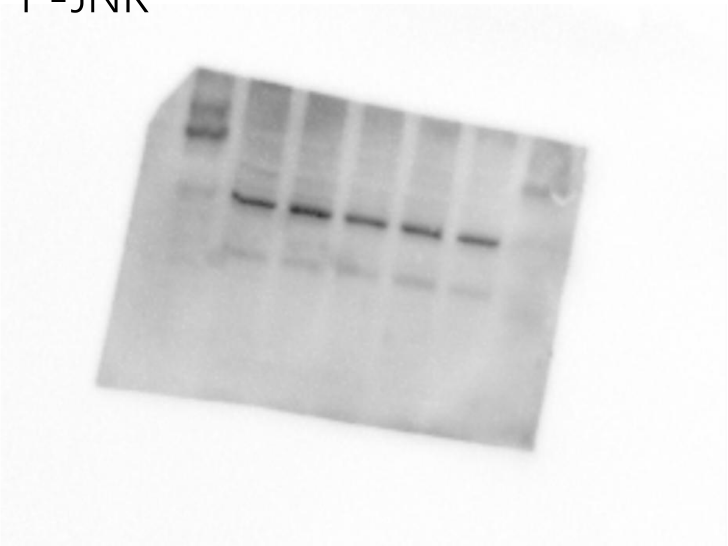

JNK

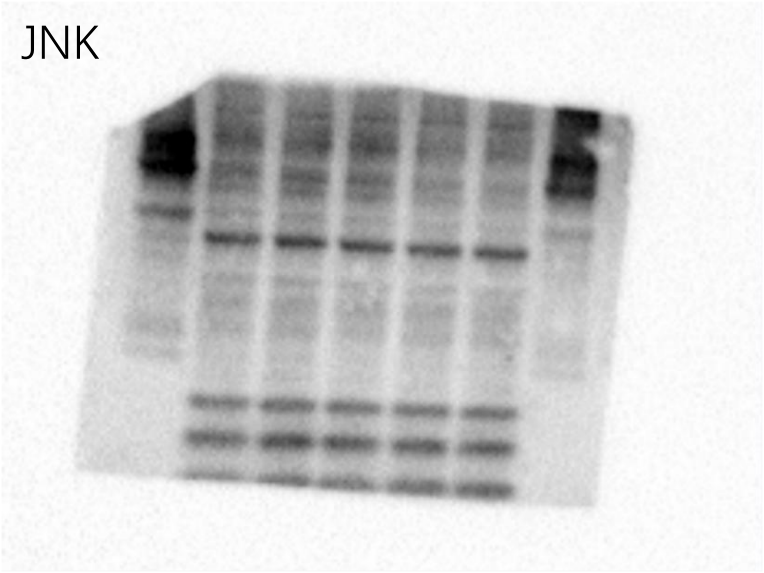

P-JNK

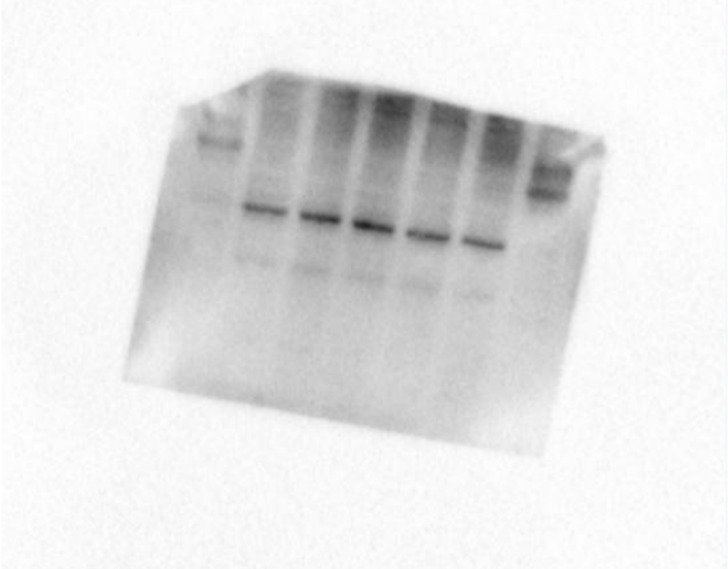

Supplement: Supplementary file 2 [file DataSheet4.pdf]
