# Supplementary material for: Sanhuang Fukang oil alleviates X-ray-induced skin injury by reducing inflammation and apoptosis: an in vivo study
Source: Front Pharmacol. 2026 Jan 6;16:1684426. doi: 10.3389/fphar.2025.1684426 (PMC12816242; doi:10.3389/fphar.2025.1684426)
Supplement: Supplementary file 3 [file DataSheet3.PDF]

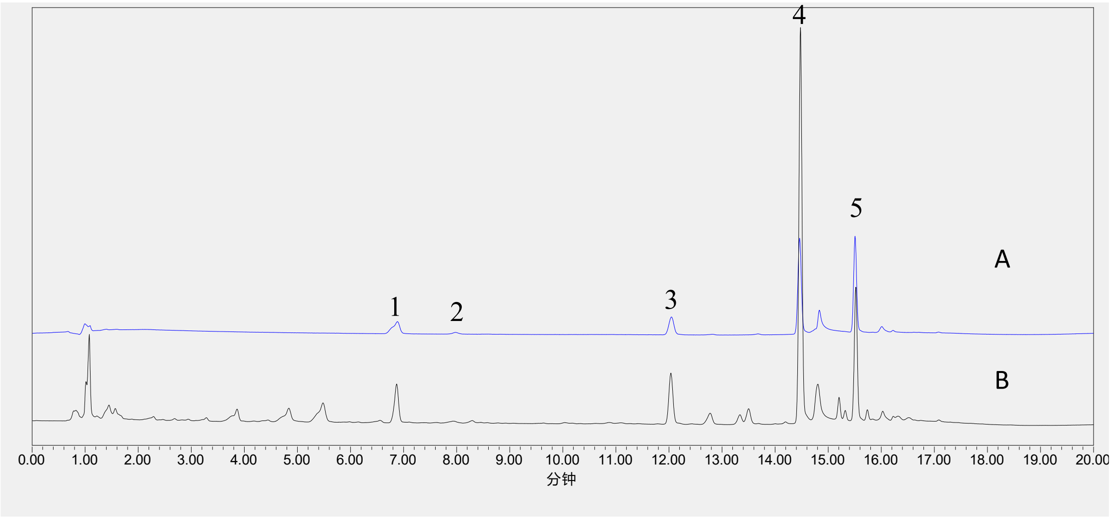

A: standards(1：Aloe emodin；2：Rhein：3：Emodin：4：Chrysophanol；5：Physcion)

B: sample

Figure 1: the chromatography of Sanhuang Fukang Oil

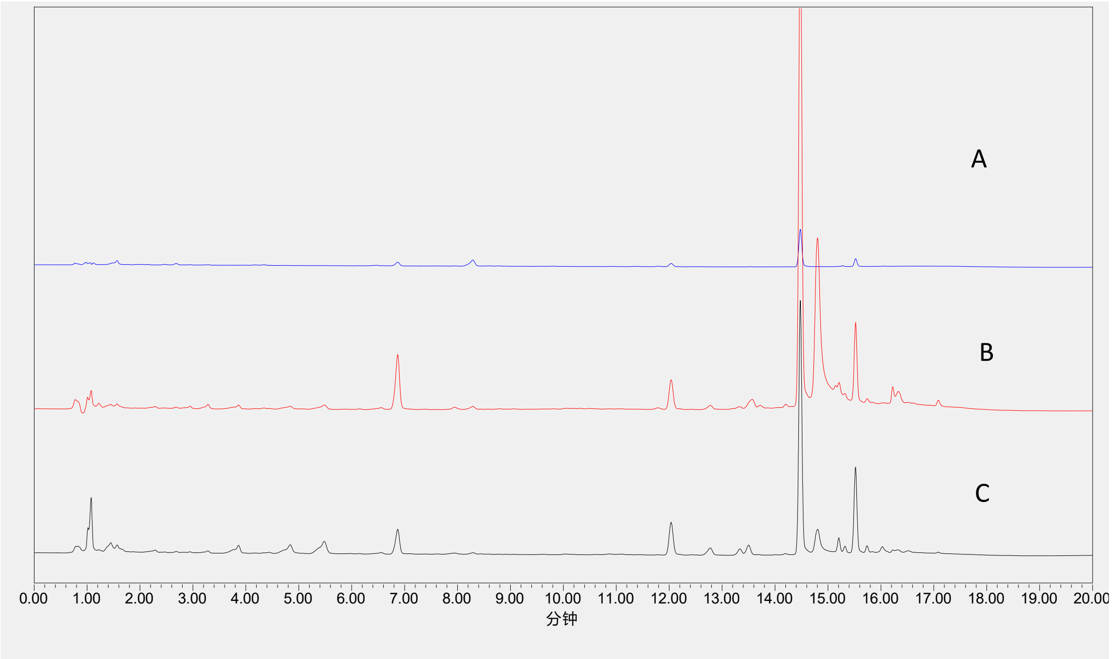

Figure 2: The fingerprint of Sanhuang Fukang Oil in three different wavelengths (A: 354nm B: 254nm C: 280nm)

Sanhuang Fukang Oil was prepared by soak method, specifically, Coptis chinensis, Rheum officinale, Phellodendron amurense, Alumen and Borneol were grinded into powder. Take 50g (10g of each) of the mixture and add it to 100ml of olive oil heated to 70-80°C. Seal and let it soak for one week. Filter the mixture through gauze to remove residues. Coptis chinensis, Rheum officinale,

Phellodendron amurense, Alumen and Borneol were supplied by pharmacy department of the 1st affiliated hospital of Guangzhou University of Chinese Medicine. Quality control of all raw materials was authenticated based on the Chinese Pharmacopoeia(National Pharmacopoeia Commission, 2020).

Table 1: composition of Sanhuang Fukang Oil

Name	Herb name	Manufactory	Dosage
Coptis chinensis	Huang Lian	Kangmei Pharmaceutical	10g
Rheum officinale Baill	Da Huang	Kangmei Pharmaceutical	10g
Phellodendron amurense Rupr	Huang Bo	Kangmei Pharmaceutical	10g
Potash alum	Ming Fan	Kangmei Pharmaceutical	10g
Synthetic Borneol	Bing Pian	Kangmei Pharmaceutical	10g

Table 2 . The stability of Sanhuang Fukang Oil

Time	Aloe emodin	Rhein	Emodin	Chrysophanol	Physcion
0 day	91427	4144	119592	688574	224806
1 day	90836	4267	118551	685726	230463
2 day	91065	4165	118605	684956	226598
3 day	91343	4271	118643	683844	222641
4 day	91135	4027	119704	690650	221908
5 day	91050	4247	129724	695681	223764
6 day	92718	4234	121834	706618	227904
7 day	92202	4009	123642	725654	235201
RSD	0.74%	2.71%	1.54%	2.13%	2.13%

The stability of Sanhuang Fukang Oil was monitored by peak area of main compounds by UHPLC. The data showed it was stable for at least 7 days.
