# Supplementary material for: Sanhuang Fukang oil alleviates X-ray-induced skin injury by reducing inflammation and apoptosis: an in vivo study
Source: Front Pharmacol. 2026 Jan 6;16:1684426. doi: 10.3389/fphar.2025.1684426 (PMC12816242; doi:10.3389/fphar.2025.1684426)
Supplement: Supplementary file 5 [file DataSheet5.pdf]

分类号 R268　　　　学校代号 10572

UDC 610 密级 公开　　　　学　　号 20201120118

广州中医药大学

Guangzhou University of Chinese Medicine

硕士学位论文

“三黄肤康油”防治乳腺癌急性放射性皮炎的疗效评价与机制探讨

学位申请人　黄佳榕

指导教师姓名　黄梅

专业名称　中医外科学

申请学位类型　专业学位

论文提交日期　2023 年 6 月

中国知网 https://www.cnki.net

// 摘　要

目的：

在前期实验及临床研究的基础上，进一步通过临床随机对照试验，评价“三黄肤康油”对乳腺癌患者放疗期间皮损情况及生活质量的影响；通过网络药理学初步探讨“三黄肤康油”防治急性放射性皮炎的作用机制；建立放射性皮肤损伤细胞模型，为后续网络药理学验证奠定基础。

方法：

1. 采用前瞻性随机对照研究，纳入 2020 年 7 月至 2022 年 9 月就诊于广州中医药大学第一附属医院放疗科的 180 例接受放疗的乳腺癌患者，通过分层区组随机化法，按不同放疗剂量分割方案分别分为 A、B、C 三层，再 1:1 随机分配到“三黄肤康油”试验组和“奥克喷”对照组。两组患者均从放疗首日起每日外用药物直至放疗全部完成后第 21 天。分别在用药前，用药第 14、21 天及用药结束后评估 RTOG/EORTC 分级及 DLQI 评分，并在用药结束后评估急性放射性皮炎防治总疗效。

2. 在 HERB 数据库中获取三黄肤康油的主要化学成分，经过药代动力学筛选后将有效成分上传至 SwissTargetPrediction 平台预测相关靶点，与疾病数据库获取的放射性皮炎相关靶点相交后获得交集靶点，上传至 STRING 平台构建蛋白质互作网络，同时上传至 Metascape 平台进行富集分析，根据富集分析结果构建药物成分-靶点-通路图，筛选出核心靶点及成分，进行分子对接。

3. 用 0、2.5、5、7.5、10、15、20Gy 的 X 射线照射 HaCaT 细胞，分别在照射后 24、48、72h 使用 CCK-8 法检测 HaCaT 细胞活力并进行形态学观察，利用克隆形成实验检测细胞克隆形成能力，划痕实验观察 HaCaT 细胞迁移能力，Western blot 检测 p-Akt 蛋白表达水平，从而确定 HaCaT 细胞放射性皮肤损伤模型的造模条件。

结果：

1. 两组受试者急性放射性皮炎的总发生率分别为 48.78%和 65.00%，差异具有统计学意义（P=0.037）。两组受试者 RTOG/EORTC 分级在用药第 14、21 天差异不具有统计学意义（P=0.541，P=0.244），在用药结束时两组整体差异具有统计学意义（P=0.016）。两组急性放射性皮炎防治总有效率分别为 98.78%和 97.50%，差异无统计学意义（P=0.618）。两组受试者用药前至用药后 21 天 DLQI 评分秩平均值呈逐渐升高趋势，P<0.001，差异具有统计学意义。用药第 14、21 天试验组 DLQI 评分分别为 0（0，1.25）分、1（0，3）分，均低于对照组的 1（0，4）、1.5（0，4）分，组间差异具有统计学意义（P<0.05）。

2. 网络药理学预测三黄肤康油防治放射性皮炎的主要化学成分可能为山奈酚、槲皮素、白杨素、黄芩素、染料木黄酮，主要的作用靶点可能为 AKT1、PIK3R1、PIK3CA、PIK3CB、ESR1、EGFR，所涉及的信号通路主要包括癌症通路、PI3K-Akt 信号通路、

中国知网 https://www.cnki.net

化学致癌-活性氧通路等。以上成分与靶点分子对接结合能均小于-5kcal/mol。

3. 形态学观察发现，与0Gy对照组相比，吸收剂量越高的组别细胞增殖越慢，细胞形态改变越明显。CCK-8法检测细胞存活率，除10Gy组24h检测值外，余各组细胞存活率均与同时间点对照组有明显的统计学差异，$P<0.05$。5Gy照射48h后，细胞存活率为（41.79±5.06）%。照射剂量≥5Gy时，细胞克隆形成率相较于0Gy对照组明显降低，$P<0.05$，差异具有统计学意义。照射后HaCaT细胞划痕愈合率较对照组均有不同程度降低，除2.5、20Gy组，其余各照射剂量组与对照组相比，$P<0.05$，差异均具有统计学意义。

结论：

1. 三黄肤康油能有效防治乳腺癌放射性皮炎，其疗效不劣于奥克喷；三黄肤康油能降低乳腺癌患者放疗期间DLQI评分，有效提高生活质量，效果优于奥克喷。

2. 三黄肤康油可能主要通过山奈酚、槲皮素、白杨素、黄芩素、染料木黄酮等主要成分干预癌症通路、PI3K-Akt信号通路、化学致癌-活性氧通路等发挥治疗放射性皮炎的作用。

3. X射线照射使HaCaT细胞细胞活力、细胞增殖能力和细胞迁移能力减弱，照射剂量为5Gy的X线照射后孵育48h是HaCaT细胞放射性皮肤损伤适宜的造模条件。

关键词： 放射性皮炎；乳腺癌；三黄肤康油；网络药理学；细胞实验

中国知网 https://www.cnki.net

The impact and mechanism of "Sanhuang Fukang Oil" on the quality of life in patients with acute radiation dermatitis in breast cancer

Specialty: Surgery of traditional Chinese medicine
Author: Jiarong Huang
Tutor: Mei Huang

Abstract

Objective

Based on previous experiments and clinical studies, this study aims to evaluate the impact of "Sanhuang Fukuang Oil" on the quality of life of breast cancer patients during radiotherapy through a clinical randomized controlled trial. Additionally, the study aims to explore the mechanism of "Sanhuang Fukuang Oil" in preventing and treating acute radiation dermatitis through network pharmacology. To lay the foundation for subsequent network pharmacology validation.

Methods

1. A prospective randomized controlled study was conducted, involving 180 breast cancer patients who received radiotherapy at the Radiotherapy Department of the First Affiliated Hospital of Guangzhou University of Chinese Medicine from July 2020 to September 2022. The patients were divided into three groups, A, B, and C, according to different radiotherapy dose fractionation schemes, using a stratified randomization method. Within each group, the patients were randomly assigned in a 1:1 ratio to either the "Sanhuang Fukang Oil" experimental group or the "Aqueous Cream" control group. Both groups of patients applied the topical medication daily from the first day of radiotherapy until the completion of the full course, which lasted 21 days. The RTOG/EORTC grading and DLQI (Dermatology Life Quality Index) score were assessed before medication, on the 14th and 21st day of medication, and at the end of medication. The overall therapeutic effect in the prevention and treatment of acute radiation dermatitis was evaluated after the completion of medication.

2. The main chemical components of "Sanhuang Fukuang Oil" were obtained from the HERB database, and the effective components were screened through pharmacokinetics . The effective components were uploaded to the "Swiss Target Prediction" platform to predict related targets. The disease targets obtained from the disease database were then taken to obtain the intersection target. The intersection targets were uploaded to the STRING platform to construct a protein interaction network and the Metascape platform for enrichment analysis. Based on the results of the enrichment analysis, a drug component-target-pathway diagram was constructed, which core targets and components were screened from it. Finally, perform molecular docking by using these core targets and

中国知网 https://www.cnki.net

components.

3. HaCaT cells were irradiated with X-rays at doses of 0, 2.5, 5, 7.5, 10, 15, and 20 Gy, and cell viability was detected using the CCK-8 assay and morphological observations were made at 24, 48, and 72 hours after irradiation. The cell clonogenic formation ability was detected using a cloning formation experiment, while the cell migration ability was detected using a scratch test. The expression level of p-Akt protein was detected by Western blot to determine the modeling conditions for the radiation-induced skin injury model in HaCaT cells.

Results

1. There was no significant difference in RTOG/EORTC grading between the two groups on day 14 and day 21 of medication (P=0.541, P=0.244), but there was a significant difference at the end of medication (P=0.016). The overall effective rates of prevention and treatment of acute radiation dermatitis in the two groups were 98.78% and 97.50%, respectively, with no significant difference (P=0.618). The ranks of DLQI scores from pre-medication to 21 days post-medication showed a gradual increase in both groups, with a significant difference (P<0.001). The DLQI scores of the experimental group on day 14 and day 21 were 0 (0, 1.25) and 1 (0, 3) points, respectively, which were lower than the control group (1 (0, 4) and 1.5 (0, 4) points, respectively), with statistically significant differences between the groups (P<0.05). The total incidence rates of acute radiation dermatitis in the two groups were 48.78% and 65.00%, respectively, with a significant difference (P=0.037).

2. Network pharmacology predicts that the main chemical components of "Sanhuang Fukuang Oil" for the prevention and treatment of radiation dermatitis may be quercetin, kaempferol, baicalein, chrysin, and genistein. The main target molecules involved are AKT1, PIK3R1, PIK3CA, PIK3CB, ESR1, and EGFR, and the signaling pathways involved mainly include cancer pathways, the PI3K-Akt signaling pathway, the epidermal growth factor receptor tyrosine kinase inhibitor resistance pathway, and the chemical carcinogenesis-reactive oxygen species pathway. The binding energies between the above components and target molecules are all less than -5 kcal/mol.

3. Morphological observations revealed that the higher the absorbed dose, the slower the cell proliferation and the more significant the changes in cell morphology compared to the 0Gy control group. CCK-8 assay showed that except for the 24-hour survival rate in the 10Gy group, there was a significant statistical difference in cell survival rates compared to the control group at the same time point (P < 0.05). The cell survival rate was (41.79 ± 5.06)% 48 hours after 5Gy irradiation. The cell clone formation rate was significantly lower in groups irradiated with a dose of ≥5Gy compared to the 0Gy control group (P < 0.05). The scratch healing rate of HaCaT cells after irradiation was significantly reduced compared to the control group, except for the 2.5 and 20Gy groups, and the differences were statistically significant (P < 0.05).

Conclusion

中国知网 https://www.cnki.net

1. Sanhuang Fukang Oil demonstrates effective prevention and treatment of radiation dermatitis in breast cancer, with non-inferior efficacy compared to "Oak spray". Sanhuang Fukang Oil can reduce the DLQI score during radiotherapy in breast cancer patients, effectively improving their quality of life, with better efficacy than "Oak spray".

2. "Sanhuang Fukuang Oil" may mainly intervene in cancer pathways, PI3K-Akt signaling pathway, epidermal growth factor receptor tyrosine kinase inhibitor resistance pathway, chemical carcinogenesis-reactive oxygen species pathway, and other signaling pathways through its main ingredients such as quercetin, kaempferol, baicalein, chrysin, and genistein, to play a therapeutic role in treating radiation-induced dermatitis.

3. X-ray irradiation weakens the cell vitality, proliferation ability, and migration ability of HaCaT cells. Irradiation with a dose of 5Gy of X-rays followed by incubation for 48 hours is a suitable modeling condition for radiation skin damage in HaCaT cells.

Key words: Acute radiation dermatitis; Breast cancer; Sanhuang Fukuang Oil;
Network pharmacology; Cell experimen

中国知网 https://www.cnki.net

分类号 R268　　　　学校代号 10572

UDC 610 密级 公开　　　　学 号 20192109532

广州中医药大学

Guangzhou University of Chinese Medicine

硕士学位论文

“三黄肤康油”防治乳腺癌急性放射性皮炎的临床研究

学位申请人 龚嘉倩

指导教师姓名 黄梅

专业名称 中医外科学

申请学位类型 专业学位

论文提交日期 2022 年 6 月

中国知网 https://www.cnki.net

摘　要

目的：

在前期研究的基础上，通过前瞻性随机对照临床试验，进一步评价“三黄肤康油”防治乳腺癌放疗患者急性放射性皮炎的临床疗效及安全性。

方法：

采用非劣效性前瞻性随机对照临床研究。

1. 病例收集及随机分组：收集2020年7月至2021年9月于广州中医药大学第一附属医院放疗科住院或门诊行放射治疗且符合入选标准的乳腺癌患者共120例，通过分层区组随机化法，根据手术方式进行分层：将改良根治术后、保乳无腋清术后、保乳腋清术后分别设置为A层、B层、C层，各层例数分别为67例，41例和12例，再对每个分层进行区组随机化分组，以1：1的比例随机分配到实验组和对照组。

2. 用药方法：两组患者均从放疗首日起每天坚持外用药物防治，直到放疗全部完成后第21天，其中实验组使用“三黄肤康油”，对照组使用“奥克喷”。

3. 主要观察指标为：放疗结束后第21天临床治疗皮损的总有效率；次要观察指标为：用药过程中皮损、疼痛、瘙痒的首次出现时间、首次缓解时间，发生率及首次症状持续时间；用药第14天、21天及放疗结束后第21天疼痛、瘙痒程度及皮损程度。放疗结束后21天临床皮损治疗的显效率以及疼痛、瘙痒的缓解率和治愈率。对于出现可测量面积的具有明显边界的皮损、湿性脱皮、皮肤溃疡、坏死等非IV度皮损，于皮损出现后的第3天、第7天、第10天、第14天、第21天及放疗结束后第21天后分别进行皮损面积测量。

4. 安全性评价：用药第14天、21天及放疗结束后第21天评估用药安全性：血常规、肝功能、肾功能及记录安全性相关事件。

5. 药效经济学评价：放疗结束后第21天统计每人产生的用药总费用。

结果：

1.首要研究指标：放疗结束后第21天两组皮损治疗的总有效率：实验组总有效率为98.2%，对照组为96.5%，两组比较$P>0.05$，提示“三黄肤康油”对乳腺癌急性放射性皮炎皮损治疗疗效与奥克喷相当，但显示出一定的优效趋势。

2.次要研究指标：用药期间及放疗结束后第21天两组预防和治疗效果对比

（1）预防作用

①两组皮损及疼痛、瘙痒发生率：实验组皮损、瘙痒、疼痛发生率分别为53.7%，59.3%，53.7%，对照组的发生率分别为70.0%，75.0%，66.7%；两组比较$P>0.05$。两组疗效相当，但“三黄肤康油”对比奥克喷症状发生率更低，有优效趋势。

中国知网　https://www.cnki.net

②两组皮损及疼痛、瘙痒首次出现时间：实验组皮损、瘙痒、疼痛首次出现时间（中位数）分别为:22.00天、15.50天及8.50天；对照组分别为22.00天、16.00天及3.50天；组间比较结果无统计学差异（$P>0.05$），提示两组延缓症状出现时间方面效果相当。

（2）治疗作用

① 放疗结束后第21天瘙痒、疼痛缓解率：实验组治疗瘙痒、疼痛缓解率均为100%，对照组同样均为100%；组间比较无统计学意义（$P>0.05$）。提示“三黄肤康油”与奥克喷相比在治疗疼痛、瘙痒方面疗效相当。

② 放疗结束后第21天皮损治疗显效率及疼痛、瘙痒治愈率：实验组治疗皮损显效率为57.1%，对照组为31.6%；实验组瘙痒、疼痛治愈率分别为76.8%，85.7%，对照组分别为59.6%，61.4%，组间比较均有统计学意义（$P<0.05$），提示“三黄肤康油”与奥克喷相比其皮损治疗的疗效质量更优，能更好的治愈疼痛及瘙痒症状。

③两组皮损及疼痛、瘙痒首次缓解时间：实验组瘙痒首次缓解时间中位数为放疗第18.00天，对照组为第30.00天，组间比较有统计学差异（$P<0.05$）。提示“三黄肤康油”比奥克喷能更快的缓解首次瘙痒症状；实验组皮损、疼痛首次缓解时间中位数分别为31.50天、15.50天，对照组分别为35.50天、17.50天，组间比较均无统计学差异（$P>0.05$），结果提示两组对皮损、疼痛首次缓解效果相当，但“三黄肤康油”显示出优效趋势。

④ 两组皮损及疼痛、瘙痒首次出现后持续时间：实验组首次出现皮损、瘙痒、疼痛后的持续时间中位数分别为2.00天、0.50天、0.00天，而对照组分别为7.50天、8.00天、4.00天，组间比较均有统计学差异（$P<0.05$）；提示“三黄肤康油”对比奥克喷能更快缩短首次皮损、瘙痒及疼痛出现的持续时间。

⑤ 两组用药期间皮损及疼痛、瘙痒各级人数结构对比：

皮损：两组放疗结束后第21天皮损各级人数比例差异具有统计学意义（$P<0.05$）：其中0度皮损人数比例实验组较对照组相比更高（实验组57.1%，对照组31.6%）；而两组用药第14、21天皮损各级人数差异无统计学意义（$P>0.05$），提示放疗结束后第21天“三黄肤康油”对比奥克喷治疗皮损效果更好，恢复至无皮损的人数更多，而在用药期间两组治疗皮损效果相当。

疼痛与瘙痒：用药期间实验组对比对照组疼痛、瘙痒为0度的比例更高：实验组用药第14、21天及放疗结束后第21天的无痛率分别为94.8%，81.0%，87.5%，对照组分别为81.0%，51.6%，63.2%；实验组无痒率分别为91.4%，75.9%，83.8%，对照组分别为69.4%，53.2%，64.9%，均具有统计学意义（$P<0.05$），提示在用药期间及放疗结束后第21天“三黄肤康油”对比奥克喷治疗和缓解疼痛、瘙痒效果更好，无痛率及无痒率更高；

⑥ 两组可测量皮损面积变化对比：两组在出现可测量皮损后第3、7、10、14、

中国知网 https://www.cnki.net

21天及放疗结束后第21天皮损面积呈逐步缩小趋势，差异无统计学意义（$P>0.05$），提示两组治疗缩小可测量皮损面积效果相当。

（3）两组药效经济学对比：实验组人均用药总费用中位数为285.6元，而对照组为6360.0元，两者相差22倍，结果具有统计学差异（$P<0.05$），提示“三黄肤康油”具有明显的药效经济学优势。

（4）两组安全性对比：实验组中止研究1例；对照组中止研究5例。两组受试者在用药期间的血分析、肝肾功能均未见明显异常，组间比较均无统计学差异（$P>0.05$），提示两组均安全性较好。

（5）据手术方式及放疗方式分层对比：据分层对比，A层、B层、C层三者的预防及治疗对比均显示无统计学差异（$P<0.05$），考虑与C层纳入人数较少相关。

结论：

1. 在乳腺癌术后急性放射性皮炎的预防方面：“三黄肤康油”和奥克喷疗效相当，“三黄肤康油”有一定的优效趋势。

2. 在乳腺癌术后急性放射性皮炎的治疗方面：“三黄肤康油”与奥克喷治疗乳腺癌急性放射性皮炎总疗效相当，“三黄肤康油”在部分评价指标上显示出优效：在放疗结束后第21天总有效率更高、症状缓解率更高、能更好的改善皮损症状、更快缩短首次症状出现的持续时间。

3. “三黄肤康油”在药效经济学方面体现出显著优势，安全性好，是一种具有中医特色及广泛临床应用前景的乳腺癌急性放射性皮炎的预防用药。

关键词： 乳腺癌；急性放射性皮炎；放疗

中国知网 https://www.cnki.net

The clinical study of effect of external application "Sanhuang Skin-Healthy Oil" in the prevention and treatment of acute radiation dermatitis in breast cancer

Specialty: Surgery of TCM
Author: Gong Jiaqian
Tutor: Huang Mei

Abstract

Objective

Based on previous experiments and further through prospective randomized controlled clinical trials, we evaluated the clinical efficacy and safety of "Sanhuang Skin-Healthy Oil" in the prevention and treatment of acute radiation dermatitis in breast cancer.

Methods

A non inferiority prospective randomized controlled clinical study was used..

1. Case collection and randomization: A total of 120 patients including outpatients and inpatients with breast cancer who received radiotherapy and met the inclusion criteria were collected from the Department of radiotherapy, First Affiliated Hospital of Guangzhou University of Chinese Medicine from July 2020 to September 2021.Stratified randomized block design was adopted and divided into three layers according to three operation options: modified radical mastectomy, breast-conserving therapy without axillary lymph node dissection and breast-conserving therapy with axillary lymph node dissection, they were divided into three layers: layer a, layer B and layer C, and the number of cases in each layer is 67,41 and 12., Then each layer was randomly divided into two groups, and randomly assigned to the treatment group and control group in the ratio of 1:1.

2.Medication method: Both groups of patients insisted on external application every day from the first day of radiotherapy until the 21st day after the completion of radiotherapy.The experimental group used "Sanhuang Skin-Healthy oil" and the control group used "oakspray".

3.Primary observation index: The total effective rate of clinical treatment of skin lesions was evaluated 21 days after radiotherapy. The secondary outcome measures: The first occurrence time and first remission period of each skin lesion grade, pain and pruritus, and the incidence and first symptom duration during medication. The degree of pain, pruritus, and skin lesion were evaluated on the 14th and 21st days of treatment and the last day of medication. Clinical treatment skin lesions were evaluated 21 days after radiotherapy including effective rate of skin lesions, as well as the relief rate and cure rate of pain and pruritus.The total clinical curative effects were evaluated on the last day of medication. Except for IV skin lesions, the measurable area of skin lesions such as skin lesions with obvious boundary, wet peeling, skin ulcer and necrosis shall be measured on the 3rd, 7th,

中国知网 https://www.cnki.net

10th, 14th and 21st days after the occurrence of skin lesions and the last day of medication.

4. Safety evaluation: The drug safety (blood routine examination, liver function, renal function and security related events) was evaluated and recorded on the 14th and 21st day of the drug use and after the drug use.

5. Pharmacodynamic and economic evaluation: Count the total cost of medication per person after medication.

Results

1. Primary research index: the total therapeutic effect on curing skin lesions of the two groups on the 21th day after the end of radiotherapy. The total effective rate of skin lesions in the experimental group was 98.2%, and that in the control group was 96.5%. The *P* value of the two groups was compared($P > 0.05$). It is suggested that "Sanhuang Skin-Healthy Oil" has a equivalent therapeutic effect on the skin lesions in the treatment of acute radiation dermatitis of breast cancer with oakspray, but it shows a trend of excellent efficiency.

2. Secondary indicators: Comparison of prevention and treatment effects between the two groups during and after medication.

(1)Prevention

3. ①Incidence of skin lesions, pain and pruritus between the two groups: the incidence of skin lesions, pruritus and pain in the experimental group were 53.7%, 59.3% and 53.7% respectively, while the incidence in the control group was 70.0%, 75.0% and 66.7%. There was no significant difference ($P > 0.05$). The curative effects of the two groups were similar, however, "Sanhuang Skin-Healthy oil" had a lower incidence of symptoms than oakspray and it shows a trend of excellent efficiency.

②The first occurrence time of skin lesions, pain and pruritus between the two groups: After the start of radiotherapy, the first occurrence time (median) of skin lesions, pruritus and pain in the experimental group were 22.00 days, 15.50 days and 8.50 days respectively, while those in the control group were 22.00 days, 16.00 days and 3.50 days respectively; There was no significant difference between the two groups ($P > 0.05$), suggesting that the two groups had the same effect in delaying the occurrence time of symptoms.

(2)Treatment

①The relief rates of pruritus and pain on the 21st day after radiotherapy: the relief rates of pruritus and pain in the experimental group were 100% respectively, as well as 100% respectively in the control group. There is no statistical significance ($P > 0.05$).It is suggested that the therapeutic effect of "Sanhuang Skin-Healthy oil" is equivalent to that of oakspray.

②Effective rate and cure rate of skin lesions, pain and pruritus on the 21st day after radiotherapy::The effective rates of skin lesions in the experimental group were 57.1%, while in the control group were 31.6%. The cure rates of pruritus and pain in the experimental group were 76.8% and 85.7% respectively, and those in the control group

中国知网 https://www.cnki.net

were 59.6% and 61.4% respectively, which were statistically significant (P< 0.05), suggesting that compared with oakspray, "Sanhuang Skin-healthy oil" has better curative effect and quality in the treatment of skin lesions, and can better cure the symptoms of pain and pruritus.

③ The first remission time of skin lesions, pain and pruritus between the two groups: the median time of the first remission of pruritus in the experimental group was 18.00 days after radiotherapy and 30.00 days in the control group. The results were statistically different (P < 0.05). It is suggested that "Sanhuang Skin-Healthy oil" can alleviate the first itching faster than oak spray. The median time of first relief of skin lesions, and pain in the experimental group were 31.50 days and 15.50 days respectively, and those in the control group were 35.50 days and 17.50 days respectively; "Sanhuang Skin-Healthy oil" has faster relief time for skin lesions and pain than oak spray, but there is no significant difference between the two groups (P > 0.05), suggesting that the two groups have the same effect on the first relief of skin lesions and pain, but "Sanhuang Skin-healthy oil" shows a trend of excellent efficiency.

④The duration after the first appearance of skin lesions, pain and pruritus between the two groups: the median duration of the first skin lesion, first pruritus and first pain in the experimental group was 2.00 days, 0.50 days and 0.00 days, and that in the control group was 7.50 days, 8.00 days and 4.00 days. The results were statistically different (P < 0.05). It is suggested that "Sanhuang Fukang oil" can shorten the duration of first skin lesions, pruritus and pain faster than oak spray.

⑤Comparison of population structure at all levels of skin lesions, pain and pruritus between the two groups during medication:

Skin lesions: There was significant difference in the proportion of people at all levels of skin lesions between the two groups after the end of medication (P < 0.05). The proportion of people with 0-degree skin lesions in the experimental group was higher than that in the control group (57.1% in the experimental group and 31.6% in the control group), but there was no significant difference in the number of people at all levels of skin lesions between the two groups on the 14th and 21st days of medication (P > 0.05).It is suggested that "Sanhuang Skin-Healthy oil" is better than oakspray in the treatment of skin lesions after the end of medication, and more people recover to no skin lesions. During the medication period, the two groups have the same effect in the treatment of skin lesions.

Pain and Pruritus: During the treatment period, the proportion of pain and pruritus of 0 degree in the experimental group was higher than that in the control group. The painless rates of the experimental group on the 14th, 21st day and after the treatment were 94.8%, 81.0% and 87.5% respectively, and those of the control group were 81.0%, 51.6% and 63.2% respectively (P < 0.05) and the itch free rates of the experimental group on the 14th and 21st days and after the treatment were 91.4%, 75.9% and 83.8% respectively, and those of the control group were 69.4%, 53.2% and 64.9% respectively (P < 0.05), suggesting that

中国知网 https://www.cnki.net

"Sanhuang Skin-Healthy oil" has better pain and pruritus relief effect and higher painless rate and the rate of no pruritus is higher than oakspray during and after the treatment.

⑥ Comparison of changes in measurable lesion area between the two groups: On the 3rd, 7th, 10th, 14th, 21st days and the end of medication after the appearance of measurable lesions, the lesion area of the two groups showed a gradual reduction trend. There was no significant difference between the two groups ($P > 0.05$).It is suggested that the two groups have the same effect in reducing the measurable lesion area.

(3) Pharmaco-economics comparison between the two groups: The total cost of per person in the experimental group was median 285.6 yuan, while in the control group was 6360.0 yuan. The difference between the two groups was 22 times. The results were statistically different ($P < 0.05$).It is suggested that "Sanhuang Fukang oil" has obvious pharmacoeconomic advantages.

(4)Safety comparison between the two groups: 1 case stopped the study in the experimental group; while in the control group, 5 cases were discontinued. There was no significant abnormality in blood routine and liver and renal function between the two groups during the treatment, and there was no significant difference between the two groups ($P > 0.05$).It is suggested that both groups have good safety.

(5)Stratified comparison according to the mode of operation and radiotherapy:According to the stratified comparison, the comparison of prevention and treatment of layer a, layer B and layer C showed no statistical difference ($P < 0.05$), which was considered to be related to the small number of people included in layer C.

Conclusion

1. In the prevention of acute radiation dermatitis after breast cancer surgery: "Sanhuang Skin-Healthy oil" has the same curative effect as oak spray, "Sanhuang Skin-Healthy oil" has a certain trend of excellent effect.

2. In the treatment of acute radiation dermatitis after breast cancer surgery: "Sanhuang Skin-Healthy oil" has the same total efficacy as oakspray in the treatment of acute radiation dermatitis. Meanwhile, It shows excellent effect on some evaluation indexes with "Sanhuang Skin-Healthy oil": higher total efficiency and higher remission rate on the 21st day after radiotherapy, and it can not only improve and relieve symptoms better, but shorten the first symptom duration,

3. "Sanhuang Skin-Healthy oil" shows a significant advantage in pharmacoeconomics with good safety. It is a kind of preventive medicine for acute radiation dermatitis of breast cancer with Chinese medicine characteristics and broad clinical application prospects.

Key words： Mammary Cancer; Acute Radiation Dermatitis; Radiotherapy

中国知网 https://www.cnki.net
